# Circulating HLA-DR^+^CD4^+^ effector memory T cells resistant to CCR5 and PD-L1 mediated suppression compromise regulatory T cell function in tuberculosis

**DOI:** 10.1371/journal.ppat.1007289

**Published:** 2018-09-19

**Authors:** Asma Ahmed, Vasista Adiga, Soumya Nayak, J. Anto Jesuraj Uday Kumar, Chirag Dhar, Pravat Nalini Sahoo, Bharath K. Sundararaj, George D. Souza, Annapurna Vyakarnam

**Affiliations:** 1 Laboratory of Immunology of HIV-TB co-infection, Centre for Infectious Disease Research, Indian Institute of Science, Bangalore, India; 2 Division of Infectious Diseases, St John’s Research Institute, Bangalore, India; 3 Dept. of Pulmonary Medicine & Division of Infectious Diseases, St John’s Research Institute, Bangalore, India; 4 Department of Infectious Diseases, King’s College London, London, School of Immunology & Microbial Sciences, Faculty of Life Sciences & Medicine, Guy's Campus, London, United Kingdom; Portland VA Medical Center, Oregon Health and Science University, UNITED STATES

## Abstract

Chronic T cell activation is a hallmark of pulmonary tuberculosis (PTB). The mechanisms underpinning this important phenomenon are however, poorly elucidated, though known to rely on control of T effector cells (Teff) by regulatory T cells (Treg). Our studies show that circulating natural Treg cells in adults with PTB preserve their suppressive potential but Teff cells from such subjects are resistant to Treg-mediated suppression. We found this to be due to expansion of an activated Teff subset identified by Human Leukocyte Antigen (HLA)-DR expression. Sensitivity to suppression was restored to control levels by depletion of this subset. Comparative transcriptome analysis of Teff cells that contain HLA-DR^+^ cells versus the fraction depleted of this population identified putative resistance mechanisms linked to *IFNG*, *IL17A*, *IL22*, *PD-L1* and β-chemokines *CCL3L3*, *CCL4* expression. Antibody blocking experiments confirmed HLA-DR^+^ Teff cells, but not the fraction depleted of HLA-DR^+^ effectors, to be resistant to Treg suppression mediated via CCR5 and PD-L1 associated pathways. In the presence of HLA-DR^+^ Teff cells, activation of NFκB downstream of CCR5 and PD-L1 was perturbed. In addition, HLA-DR^+^ Teff cells expressed significantly higher levels of Th1/Th17 cytokines that may regulate Treg function through a reciprocal counter-balancing relationship. Taken together, our study provides novel insight on how activated HLA-DR^+^CD4^+^ T cells may contribute to disease associated inflammation by compromising Treg-mediated suppression in PTB.

## Author summary

An important marker of progression to PTB following *Mycobacterium tuberculosis* (*Mtb)* infection in humans is elevated frequencies of HLA-DR^+^CD4^+^ T cells, reflecting chronic T cell activation. However, the mechanisms by which activated HLA-DR^+^CD4^+^ T cells contribute to disease process is not known. We show that CD25^-^ HLA-DR^+^CD4^+^ memory Teff from PTB patients are resistant to suppression mediated by Treg cells. An unbiased transcriptome analysis identified several key pathways that contribute to this resistance. Specifically, presence of HLA-DR^+^CD4^+^ T cells renders the effector population resistant to CCR5 and PD-L1 mediated suppression by Treg cells. In addition, the HLA-DR^+^CD4^+^ memory Teff cells express elevated levels of Th1/Th17 cytokines known to counter-regulate and dampen Treg suppression. These findings provide fresh insight to disease process in TB and identify HLA-DR^+^ Teff resistant to Treg suppression as a potential functional marker of disease.

## Introduction

TB is a complex disease which claims several lives annually. In 2016, 1.3 million human immunodeficiency virus (HIV) negative (-ve) and 374,000 HIV positive (+ve) people succumbed to TB [1]. The spread of TB and mortality associated with it has been aggravated by the alarming rise in multidrug resistant (MDR) and extensively drug resistant (XDR) cases. Control of TB is further challenged by the availability of just one preventive vaccine, BCG, which when given at birth confers good protection in children but does not efficiently prevent new infection and reactivation of latent TB in adults [2]. India has the highest burden of TB and MDR TB globally. In 2016, about 2.8 million people were afflicted with TB in India, of which about 0.43 million died [3]. These statistics call for a better understanding of disease processes in *Mycobacterium tuberculosis* (*Mtb)* infection.

The functionally heterogeneous CD4^+^ T cell compartment in the body is known to be important for immunity to TB [4]. Loss of CD4^+^ T cells, induced by HIV infection, is recognised to increase susceptibility to TB and TB incidence globally [5]. Within the CD4^+^ T cell compartment, Th1 effector cytokines IFNγ and IL-12 are particularly important. Humans with mutations in genes encoding cytokines, cytokine receptors and transcription factors involved in the Th1 pathway, e.g. *IL12P40*, *STAT1*, *IFNG* and *IL12R*, are highly susceptible to *Mtb* infection and cannot even tolerate vaccination with attenuated BCG [6, 7]. Maintaining an efficacious anti-TB Th1 T effector (Teff) response is dependent on counter-regulation by an additional subset of CD4^+^ T cells, termed natural regulatory T cells (Treg). Treg cells are commonly identified by constitutive expression of CD25 (IL-2 receptor), FoxP3 (transcription factor) and reduced expression of CD127 (IL-7 receptor) and are responsible for suppressing Teff cell proliferation and cytokine expression [8]. This negative control of Teff by Treg cells is critical for immune homeostasis. Increasing evidence shows that Treg/Teff cell balance might be disrupted in *Mtb* infection. Studies in mice show that pathogen specific Treg cells expand early in *Mtb* infection and play a role in delaying the generation of IFNγ producing CD4^+^ Teff cells [9–11]. Depletion of Treg cells from *in vitro* human cultures increases IFNγ secretion in response to *Mtb* antigen stimulation [12–14]. These data indicate that Tregs may dampen protective responses and thereby hinder pathogen clearance *in vivo* [9–14]. However, the role of Treg cells in the chronic phase of TB is less clear, especially in humans. Several studies show that Tregs expand in chronic infection and may be a possible marker of disease [12–16]. However, other studies do not report such an expansion [17, 18]. More recent studies highlight a potentially important role for Treg cells in controlling TB associated inflammation. Cellular and molecular markers of immune activation are elevated in subjects with TB [19–23] or HIV-TB co-infection [24, 25], and human and animal models highlight control of such inflammation to be potentially protective [reviewed in 26]. Thus, increase in Treg frequency within granulomas of *Mtb* infected non-human primates, is interpreted as a homeostatic response to concomitant increase in CD4^+^ Teff cell numbers [27]. DBA/2 mice, which are highly susceptible to *Mtb* infection, fail to recruit sufficient FoxP3^+^ Treg cells to the lung and exhibit excessive *Mtb* induced inflammation [28], whilst IL-2 induced expansion of Tregs confers protection against *Mtb* [29].

Understanding the role of Treg cells requires not only measuring Treg frequencies, but also probing their functional characteristics. Treg mediated homeostasis is equally dependent on the potency of Treg cells to suppress Teff proliferation and cytokine expression as well as the susceptibility of Teff cells to Treg suppression. A dampened ability of Treg cells to suppress, as in many autoimmune disorders [30] or altered sensitivity of Teff cells to Treg-mediated suppression, as in HIV and Type I diabetes [31, 32], would equally disrupt T cell homeostasis. These functional characteristics of Treg-mediated immune homeostasis are poorly elucidated in TB and underpin this study. We hypothesized that maintenance of Treg-mediated homeostasis is probably disrupted in the chronic phase of *Mtb* infection and this may contribute to disease by promoting immune activation through an exaggerated Teff cell response. Our study systematically probed the quality of Treg-mediated suppression in adult PTB. We found that Treg frequency and suppressive potential are preserved in PTB. However, peripheral expansion of a subset of activated CD4^+^ Teff cells identified by HLA-DR expression compromises Treg mediated control in PTB subjects. Generalised immune activation [19–23] and expansion of activated T cells identified by high HLA-DR expression [21, 22] are well-recognised markers of TB, but, precisely how the presence of activated T cells counteracts Treg function has not been elucidated. We found that HLA-DR^+^ Teff from PTB subjects are resistant to suppression by Treg cells. RNA-Sequence analysis of Teff that contain HLA-DR^+^ cells versus the fraction depleted of this subset identified putative resistance pathways. Blocking studies show HLA-DR^+^ Teff are compromised to Treg-mediated suppression mediated through the β-chemokine receptor CCR5 as well as through the negative regulatory molecule, PD-L1. Both CCR5 and PD-L1 are reported to promote Treg suppression [33–37]; here we show for the first time their role in Teff susceptibility to Treg suppression in TB. Our data additionally highlights HLA-DR^+^ Teff to express higher levels of Th1/Th17 cytokines that might negatively regulate Treg function [38, 39]. Taken together, these data provide fresh insight into how HLA-DR^+^ Teff cells may contribute to inflammation and loss of Treg-mediated homeostasis in TB.

## Results

### Treg frequency does not vary in TB

**Fig 1:**
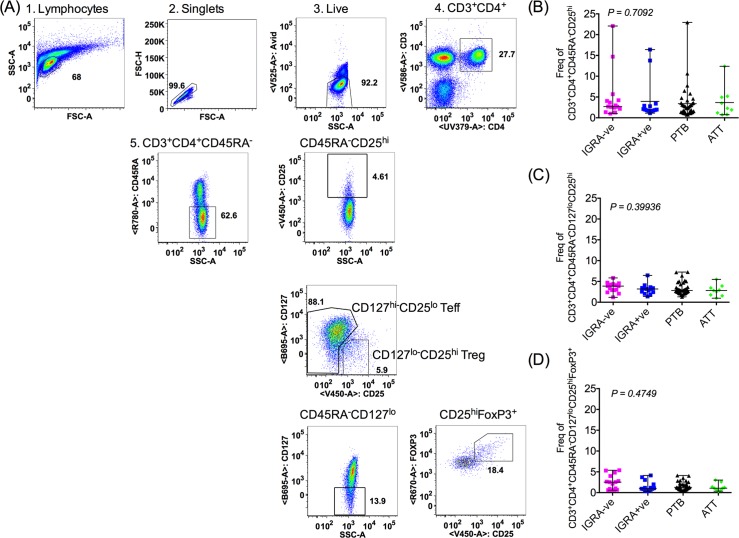
Treg frequencies are not significantly different across clinical categories. A sequential gating strategy (A) was used to define Tregs as CD3^+^CD4^+^CD45RA^-^CD25^hi^ (B), CD3^+^CD4^+^CD45RA^-^CD127^lo^CD25^hi^ (C) and CD3^+^CD4^+^CD45RA^-^CD127^lo^CD25^hi^FoxP3^+^ (D) Frequencies of all three Treg populations were calculated as frequencies of total CD3^+^CD4^+^ T cells. Data shown is median frequency with range from multiple donors (IGRA-ve N = 15, IGRA+ve N = 14, PTB N = 37, ATT 6 months N = 9) in each clinical category. P value was determined by non-parametric One-Way ANOVA Kruskal–Wallis test.

Experiments were carried out on peripheral blood mononuclear cells (PBMCs) isolated from the following groups of subjects: IFNγ release assay (IGRA) negative (IGRA-ve); IGRA positive (+ve); sputum positive, untreated pulmonary tuberculosis (PTB) and PTB subjects who have completed 6 months of anti-tubercular treatment (ATT) (Clinical summary in Table 1 and details in S1 Table). We first measured Treg frequencies across all clinical categories (Fig 1). Treg cells were characterised in three ways, using a combination of conventional markers (CD25^hi^, CD127^lo^, FoxP3^hi^) on CD4 memory T cells: CD3^+^CD4^+^CD45RA^-^CD25^hi^ (Fig 1B), CD3^+^CD4^+^CD45RA^-^CD127^lo^CD25^hi^ (Fig 1C) or CD3^+^CD4^+^CD45RA^-^CD127^lo^CD25^hi^FoxP3^+^ (Fig 1D) by following a sequential gating strategy (Fig 1A). Data in Fig 1 show frequencies of Treg cells did not vary between the different clinical categories, irrespective of the combination of markers used to define these cells (Fig 1B–1D). The frequencies of circulating peripheral Treg remain unchanged in latent and active TB disease and after ATT compared to healthy controls.

**Table 1:** Summary of subjects recruited under each clinical category.

Clinical Category	N	Median Age	Tests Performed				
			Quantiferon Gold TB Test	Sputum Acid-Fast Bacilli (AFB)	GeneXpert *Mtb*	GeneXpert Rifampicin (RIF) Resistance	Chest X-ray
IGRA-ve	16	28.5	Negative	Not performed	Not performed	Not performed	Not performed
	(9F/7M)						
IGRA+ve	14	32.5	Positive	Not performed	Not performed	Not performed	Not performed
	(3F/11M)						
PTB	45	37	Not performed	Positive	38 Positive, 3 Negative, Not performed for 4	35 Negative, 3 Positive, and Not performed for 7	22 Abnormal, 2 Normal, and not performed for 21
	(9F/36M)						
ATT 6 months	9	30	Not performed	Negative	6 Negative and 3 Detected medium to low	1 Positive, 8 Negative	Not performed for 8, Abnormal for 1
	(5F/4M)						

### Treg function is markedly compromised in active pulmonary but not latent TB

**Fig 2:**
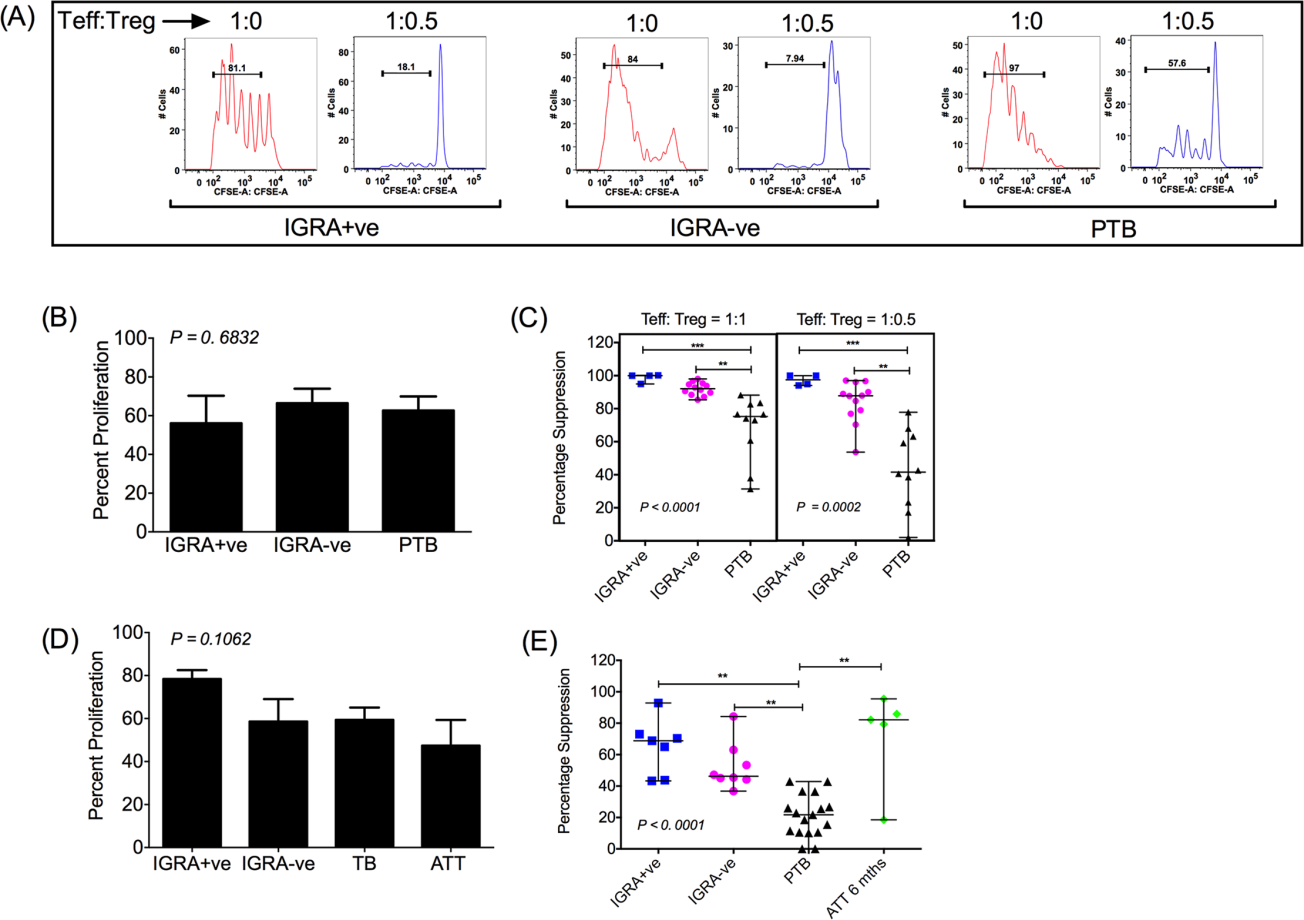
Treg mediated suppression is lower in polyclonally activated CD4^+^ Teff cells in PTB. Teff and Treg populations were isolated either by magnetic beads or flow cytometry cell sorting. Data shown in A–C is from experiments carried out using Teff and Treg cells isolated by magnetic beads and that shown in D–E is from experiments carried out using Teff and Treg cells isolated by flow cytometry sorting. CFSE labelled Teff cells were co-cultured with Treg cells at a 1:0.5 or 1:1 (C) or 1:1 (E) ratio. Cells were activated with mitogenic anti-CD3/anti-CD28 beads at a bead:Teff cell ratio of 2:1 (A–C) or 1:1 (D–E). Proliferation was measured by CFSE dilution after 4 days of culture. Representative FACS plots of Teff cell CFSE dilution in presence or absence of Treg cells from different clinical categories are shown in (A). Percentage suppression was calculated as described in Materials and Methods and is compared between different categories (C, E). Proliferation of Teff cells in absence of Tregs was compared among all clinical categories used and is shown in B and D. Data shown in B and D is mean + SEM and in C and E is median frequency/range. All data shown is from multiple donors in each category: IGRA-ve N = 12 (B, C) and 8 (D, E); IGRA+ve N = 4 (B, C) and 7 (D, E); PTB N = 10 (B, C) and 17 (D, E); ATT 6 months N = 5 (D, E). P value was determined by non-parametric One-Way ANOVA Kruskal–Wallis test and Dunn’s multiple comparisons test. ***p < 0.001, **p < 0.01, *p < 0.05.

Treg cells isolated by two approaches–(1) magnetic beads and (2) sorting by flow cytometry, were tested in functional assays. CD3^+^CD4^+^CD45RA^-^CD25^hi^ were isolated with magnetic beads and used as suppressors in functional assays. Similarly, CD3^+^CD4^+^CD45RA^-^CD127^lo^CD25^hi^ cells were isolated by flow cytometry cell sorting and used in functional assays. Both populations are reported to have clear suppressor potential [8]. CD4^+^CD45RA^-^ memory cells isolated by magnetic beads and CD3^+^CD4^+^CD45RA^-^CD127^hi^CD25^lo^ memory cells isolated by flow cytometry cell sorting, henceforth referred to as Teff were used as responders. Fig 2A–2C shows suppression mediated by CD3^+^CD4^+^CD45RA^-^CD25^hi^ Treg cells purified using magnetic beads whilst Fig 2D and 2E shows suppression mediated by CD3^+^CD4^+^CD45RA^-^CD127^lo^CD25^hi^ Treg cells isolated by cell sorting. Controls included Teff cultured without suppressors stimulated with anti-CD3/anti-CD28. Fig 2B and 2D show comparable proliferation of carboxyfluorescein succinimidyl ester (CFSE) labelled Teff cells isolated by either protocol from all subject groups tested. Further, viability of activated Teff cells from all clinical groups as measured by uptake of viability dye Avid (fixable Aqua dead cell stain) was ≥ 90% and comparable (S1 Fig). A standard 4-day suppression assay was used comprising CFSE labelled Teff cells co-cultured with autologous Treg cells with proliferation measured by CFSE dilution. Representative FACS plots of CFSE dilution in Teff from different clinical categories in absence (Teff:Treg = 1:0) or presence of autologous Treg (Teff:Treg = 1:0.5), isolated by magnetic beads, are shown in Fig 2A. Dose response curves show an expected titration of suppression over a range of Teff:Treg cell ratios in cells isolated from PTB, IGRA-ve and IGRA+ve subjects (S2 Fig), with minor differences between the two approaches of cell separation that fall within expected variation, linked possibly to inherent differences between the separation procedures as well as donor variation. A comparison of the levels of suppression among the three clinical categories showed 2- fold lower suppression in Teff cells from PTB donors (median suppression = 42.64%; range = 1.989–77.81%) compared to Teff cells from IGRA-ve healthy individuals (median suppression = 87.91%; range = 70.33–97.02%) and IGRA+ve donors (median suppression = 97.46%; range = 94.03–100%) at Teff: Treg ratio of 1:0.5 (Fig 2C) using magnetic bead sorted cells. Suppression assays performed using flow cytometry sorted cells confirmed these data, showing 3-fold lower suppression in subjects with PTB (median suppression = 21.74%, range = 0–42.94%) compared to healthy control IGRA-ve (median suppression = 53.38%, range = 44.25–84.29%) and IGRA+ve (median suppression = 73.02%, range = 43.31–92.91%) subjects at Teff: Treg ratio of 1:1 (Fig 2E). Thus, suppression of cells from subjects with TB was significantly impaired, and barely detectable in some cases using flow sorted cells, even at high Treg: Teff ratios (Fig 2E). Therefore, the difference in Treg mediated suppression between control and PTB subjects was more pronounced in assays using flow sorted cells (Fig 2E for flow sorted cells ‘vs’ Fig 2C for bead sorted cells). This is consistent with other studies that show CD3^+^CD4^+^CD45RA^-^CD127^lo^CD25^hi^ cells express higher levels of FoxP3 [40], which might contribute to the pronounced suppression in our assay. Importantly, loss of suppression in PTB was not linked to poor proliferative capacity or altered viability of Teff cells from these subjects (Fig 2B and 2D and S1 Fig). Taken together, these data highlight significant impairment of Treg-mediated suppression in PTB patients, which can be reversed by 6 months of ATT (median suppression = 82.19%, Range = 18.48–95.49%) to levels observed in healthy controls (Fig 2E).

In addition to polyclonal activation with anti-CD3/anti-CD28, we probed Treg-mediated suppression to antigen-specific stimulation (S3 Fig). Purified CD3^+^CD4^+^CD45RA^-^CD127^lo^CD25^hi^ Treg cells and PBMCs depleted of Treg cells from IGRA-ve and PTB subjects were co-cultured and activated with whole *Mtb* H37Rv lysate and IFNγ was measured by ELISA in the cell-free culture supernatant 4 days later (S3A Fig). Control cultures of PBMC depleted of Treg cells from both PTB and IGRA-ve subjects stimulated with *Mtb* lysate showed clear induction of IFNγ release (S3A Fig) with differences between the two groups likely reflecting differences in frequencies of antigen specific cells. However, at 1:1 Treg: Teff ratio, the degree of suppression in PTB cultures was significantly lower than that noted in IGRA-ve cultures (S3B Fig). These data recapitulate our observations made using anti-CD3/anti-CD28 for activation (Fig 2), confirming both antigen-specific and polyclonal Treg-mediated suppression of PTB subjects to be significantly impaired compared to that of healthy controls.

### Heterologous co-culture or criss–cross Treg suppression assay highlights Teff cells of PTB patients to be resistant to Treg suppression

**Fig 3:**
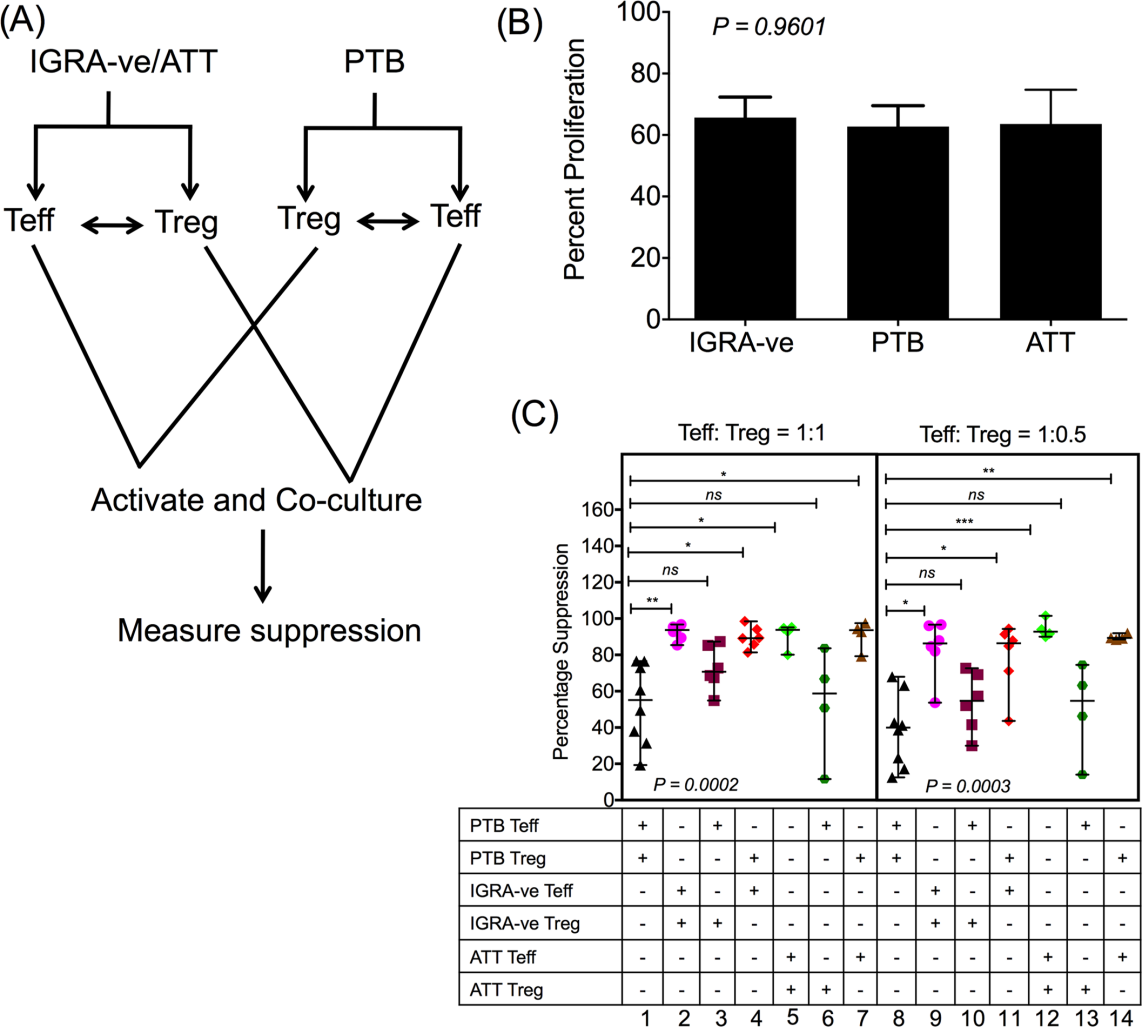
Teff cells from PTB patients are resistant to suppression whilst their Treg cells preserve suppressive potential. A diagrammatic representation of a criss-cross assay (A). A fixed number of CFSE labelled CD25^-^ effector cells isolated by magnetic beads were cultured with different ratios of either autologous or heterologous CD25^+^ suppressors also isolated by magnetic beads. Cells were activated with anti-CD3/anti-CD28 activator beads at a Teff cell:bead ratio of 2:1 for a period of 4 days. After this CFSE dilution was measured by flow cytometry. Proliferation of IGRA-ve, PTB and ATT Teff cells in absence of suppressors was compared (B). Percentage suppression was calculated as described in Materials and Methods and is compared between autologous and heterologous criss-cross cultures between Teff and Treg cells from different clinical categories (C). Cells from 6 IGRA-ve, 8 PTB and 4 ATT donors were used to set up autologous or heterologous criss-cross assays. Data shown in (B) is mean + SEM and in (C) is median and range from multiple donors. P value was determined by non-parametric One-Way ANOVA Kruskal–Wallis test and Dunn’s multiple comparisons test. ***p < 0.001, **p < 0.01, *p < 0.05, ns- not significant.

To dissect the individual roles played by Treg and Teff cells in the impaired suppression profile observed in PTB patients, a criss-cross/cross-over suppression assay was performed where IGRA-ve and ATT Tregs were co-cultured with PTB Teff and vice versa (see Fig 3A for assay plan) as previously described [41–43]. Control proliferation of IGRA-ve, PTB and ATT Teff cells in absence of suppressors (1:0 Teff:Treg ratio) was comparable (Fig 3B). Treg mediated suppression in criss-cross/cross-over reactions was compared to autologous suppression observed in PTB (column 1 and 8, Fig 3C) and IGRA-ve (column 2 and 9, Fig 3C) donors. Fig 3C shows that Treg cells isolated from PTB patients suppress heterologous IGRA-ve Teff cells as efficiently as allogeneic/autologous Teff cells (please compare columns 1 and 4; and columns 7 and 11). However, the capacity of competent Treg cells from IGRA-ve subjects to suppress Teff of PTB patients was significantly compromised (please compare columns 1 and 3; and columns 8 and 10, Fig 3C), despite the fact that the same Treg cells could effectively suppress allogeneic IGRA-ve Teff cells (columns 2 and 9, Fig 3C). In addition, we performed criss-cross assays between cells from PTB and ATT subjects. Autologous ATT suppression was significantly higher than autologous PTB suppression (please compare columns 1 and 5; and columns 8 and 12, Fig 3C). As observed with IGRA-ve cells, ATT Tregs could not suppress PTB Teff efficiently (please compare columns 1 and 6; and columns 8 and 13, Fig 3C) but PTB Treg could efficiently suppress ATT Teff (please compare columns 1 and 7; and columns 8 and 14, Fig 3C). These data reveal that despite having functional Treg cells similar to that of IGRA-ve controls and individuals having undergone ATT, PTB patients have Teff cells that are resistant to Treg mediated suppression. Thus, reduced suppression noted in Treg/Teff co-cultures from PTB donors (Fig 2) is due to altered Teff cells rather than dysfunctional Treg cells.

### PTB patients have elevated frequencies of activated Teff cells

**Fig 4:**
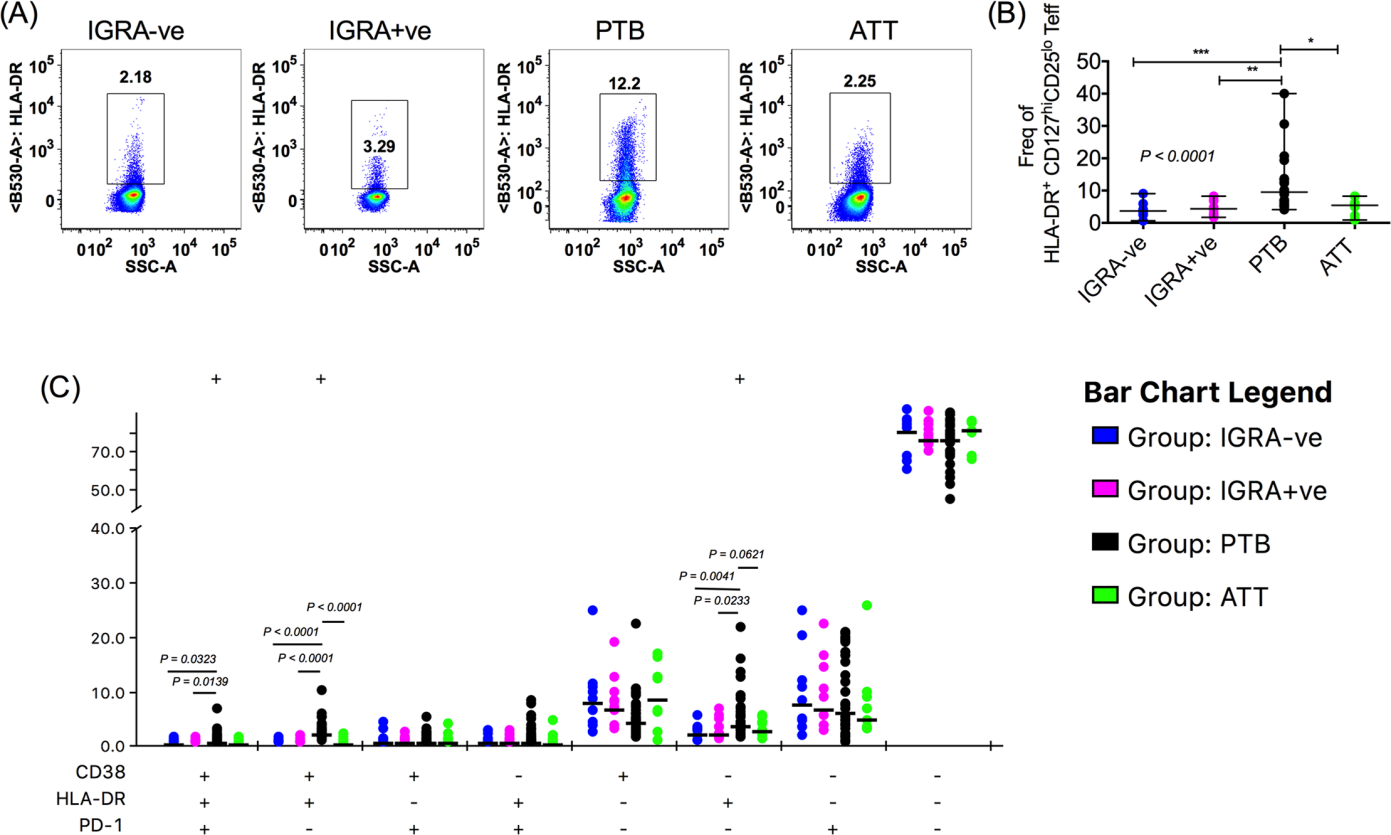
Frequency of HLA-DR^+^ Teff cells is elevated in PTB. PBMC were stained with Avid, anti-CD3, anti-CD4, anti-CD45RA, anti-CD127, anti-CD25 and anti-HLA-DR, anti-CD38 and anti-PD-1. Stained samples were acquired on a FACS Aria Fusion after using appropriate single color compensation controls. A sequential gating strategy was employed to arrive at live CD3^+^CD4^+^CD45RA^-^CD127^hi^CD25^lo^ Teff cells. Expression of HLA-DR, CD38 and PD-1 was studied in the Teff fraction. Representative HLA-DR staining on Teff cells from different clinical categories is shown (A). Frequency of HLA-DR^+^ Teff cells from different clinical categories was plotted (B). SPICE analyses of Boolean gating data of HLA-DR, CD38 and PD-1 expression on Teff cells derived from FlowJo was carried out and frequencies of Teff cells expressing different combinations of markers is shown (C). Data shown is median frequency with range from multiple donors (IGRA-ve N = 9, IGRA+ve N = 11, PTB N = 27, ATT 6 months N = 8) in each clinical category. P value for (B) was determined by non-parametric One-Way ANOVA Kruskal–Wallis test and Dunn’s multiple comparisons test. *P* value for (C) was determined by Mann Whitney test. ***p < 0.001, **p < 0.01, *p < 0.05.

One important mechanism that may contribute to Teff cells becoming resistant to Treg mediated suppression is their activation status, reflected by elevated expression of established T cell activation markers, CD69 and HLA-DR [44]. Recent studies show expansion of HLA-DR^+^ and CD38^+^ CD4^+^ T cells in PTB and HIV-TB co-infection [21, 22, 25, 45]. Elevated HLA-DR on CD4^+^ T cells was found to positively correlate with TB disease risk in BCG vaccinated infants [22]. Beyond these activation markers, PD-1 is a key functional cell surface marker with recognized importance in immune homeostasis in TB. PD-1 and PD-L1 deficient mice succumb to disease upon infection with *Mtb* due to unchecked expansion of CD4^+^ T cells [46]. While some studies in humans found expression of PD-1 to increase in PTB and decrease with treatment [47], others found it to increase post treatment [48]. Most interestingly there has been an instance where anti-PD-1 therapy for cancer treatment reactivated tuberculosis [49]. We therefore probed expression of all three of these markers on memory Teff cells (Fig 4, S4 Fig) and on Treg cells (S5 Fig).

On memory Teff cells, the most striking difference was in the frequency of HLA-DR expressing cells. Representative FACS plots of frequency of HLA-DR^+^ memory Teff (CD3^+^CD4^+^CD45RA^-^CD127^hi^CD25^lo^) cells from different clinical categories is shown in Fig 4A. HLA-DR^+^ Teff cell frequency in PTB subjects (median frequency = 9.5%, Range = 4.12–40%) was 2–2.6 fold higher compared to that of IGRA-ve controls (median frequency = 3.67%, range = 0.66–9.06%) or IGRA+ve individuals (median frequency = 4.23%, range = 1.710–8.27%) (Fig 4B) and reverted post ATT to levels seen in controls (median frequency = 5.455%, range = 0.8770–8.290%) (Fig 4B). In contrast, CD38^+^ and PD-1^+^ Teff frequencies did not vary significantly between the clinical groups (S4A–S4D Fig). On Treg cells, HLA-DR expression was elevated in PTB compared to IGRA+ve controls (S5A Fig) and CD38 was elevated in PTB with respect to IGRA-ve as well as IGRA+ve controls (S5B Fig). These elevated frequencies did not revert to baseline with ATT (S5A and S5B Fig). Comparable levels of PD-1 expression on Treg cells were observed across all clinical groups (S5C Fig).

A poly-functionality analysis using SPICE was undertaken to determine co-expression of HLA-DR, CD38 and PD-1 on Teff cells. The frequency of cells expressing all three of these markers; cells expressing 2 markers: HLA-DR and CD38 and cells single positive for HLA-DR were all significantly higher in PTB compared to controls (Fig 4C). This analysis revealed that circulating Teff cells in PTB subjects expressing HLA-DR comprise a mixture of cells single positive for HLA-DR, double positive for HLA-DR and CD38 (but not PD-1) and cells triple positive for HLA-DR, CD38 and PD-1. Elevated expression of HLA-DR alone on Teff cells is sufficient for discriminating PTB from healthy controls and treated individuals.

### HLA-DR^+^CD4^+^ Teff subset in PTB subjects is resistant to Treg-mediated suppression

**Fig 5:**
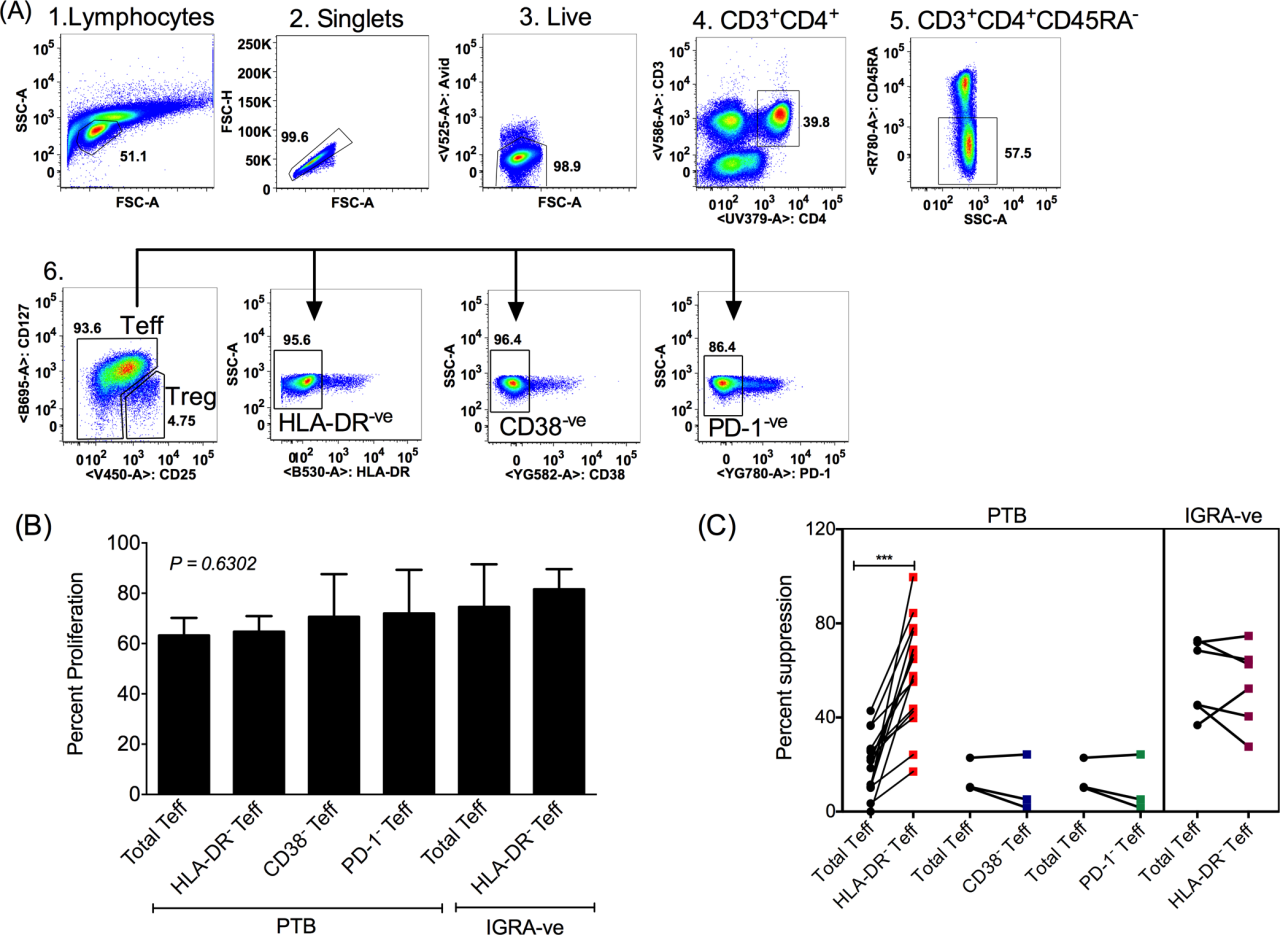
Depletion of HLA-DR^+^ fraction restores sensitivity to Treg suppression in HLA-DR^-^ Teff population from PTB subjects. A representative sequential gating strategy used for sorting pure total Teff, HLA-DR^-^, CD38^-^, PD-1^-^ Teff and Treg cells from PTB patients (A). Proliferation of Teff cells in absence of Tregs in all cell populations studied was compared (B). CFSE labeled total Teff, HLA-DR^-^, CD38^-^ and PD-1^-^ Teff cells were co-cultured with autologous Treg cells at a ratio of 1:1 in the presence of anti-CD3/anti-CD28 activator beads at a Teff:bead ratio of 1:1. Proliferation was measured by CFSE dilution after 4 days of culture and percentage suppression was calculated (C). Suppression observed in control IGRA-ve total and HLA-DR^-^ Teff cells was used for comparison (C). Total Teff and HLA-DR^-^ Teff cells were collected from 15 PTB donors and CD38^-^ and PD-1^-^ Teff cells were collected from 3 PTB donors. Data for IGRA-ve suppression was from 5 donors. Data shown in (B) is mean + SEM. P value for (B) was determined by non-parametric One-Way ANOVA Kruskal–Wallis test and Wilcoxon matched-pairs signed rank with Bonferroni correction was performed for (C). *** p < 0.001.

We hypothesized that the fraction of HLA-DR^+^ Teff cells in PTB, representing activated Teff, may contribute to resistance to Treg-mediated suppression (Fig 3C). HLA-DR^+^ cells were therefore depleted from the Teff cell pool of TB patients by cell sorting. The capacity of Treg cells to suppress Teff comprising HLA-DR^+^ and HLA-DR^-^ populations (henceforth referred to as total Teff) ‘vs’ HLA-DR^+^ depleted Teff cells (henceforth referred to as HLA-DR^-^ Teff) from PTB patients were compared. Similarly, CD38^+^ and PD-1^+^ cells were also depleted from the Teff compartment (depleted population was denoted by CD38^-^ and PD-1^-^ Teff respectively) by cell sorting (Fig 5A) and tested in suppression assays. All Teff populations studied had comparable levels of proliferation in absence of Treg cells (Fig 5B). We next tested if depletion of HLA-DR^+^, CD38^+^ and PD-1^+^ Teff cells restored suppression in cells from PTB subjects. Depletion of CD38^+^ and PD-1^+^ cells did not improve PTB Teff cell sensitivity to suppression (Fig 5C). However, depletion of HLA-DR^+^ cells significantly improved suppression and restored it to levels comparable to that seen in IGRA-ve healthy controls (Fig 5C). Depletion of HLA-DR^+^ cells from IGRA-ve individuals did not have any effect on Treg mediated suppression (Fig 5C). To further demonstrate that the HLA-DR^+^ fraction is resistant to Treg mediated suppression, we sorted total, HLA-DR^-^ and HLA-DR^+^ Teff cells and co-cultured these with autologous Treg cells in the presence of anti-CD3/anti-CD28 activator beads (S6 Fig). As the total numbers of purified HLA-DR^+^ Teff recovered from a given donor is low (median frequency 9.5% of Teff are HLA-DR^+^, please see Fig 4B), a sensitive 4-day IFNγ release assay was used to measure Treg suppression. Consistent with data in Fig 5C, suppression of purified HLA-DR^-^ Teff was significantly higher than total (comprising a mix of largely HLA-DR^-^ and a small fraction of HLADR^+^ cells) and purified HLA-DR^+^ Teff cells (S6 Fig). In addition, we also measured suppression of antigen specific stimulation (S7 Fig). PBMCs depleted of HLA-DR^+^CD4^+^ T cells (denoted as HLA-DR^-^CD4^+^Teff) stimulated with *Mtb* lysate were more sensitive to suppression by autologous Tregs compared to the PBMC fraction containing HLA-DR^+^CD4^+^ T cells (denoted as total CD4^+^ Teff) (S7A and S7B Fig). Together, these data confirm that presence of a small (median frequency = 9.5%, Range = 4.12–40%) expanded subset of HLA-DR^+^ Teff cells in PTB subjects is primarily responsible for the observed resistance to Treg mediated suppression.

### Unbiased whole genome analysis reveals pro-inflammatory cytokine genes to be up-regulated in total HLA-DR^+^ Teff cells and conversely genes associated with cell migration and homeostasis to be enriched in HLA-DR^-^ Teff cells

**Fig 6:**
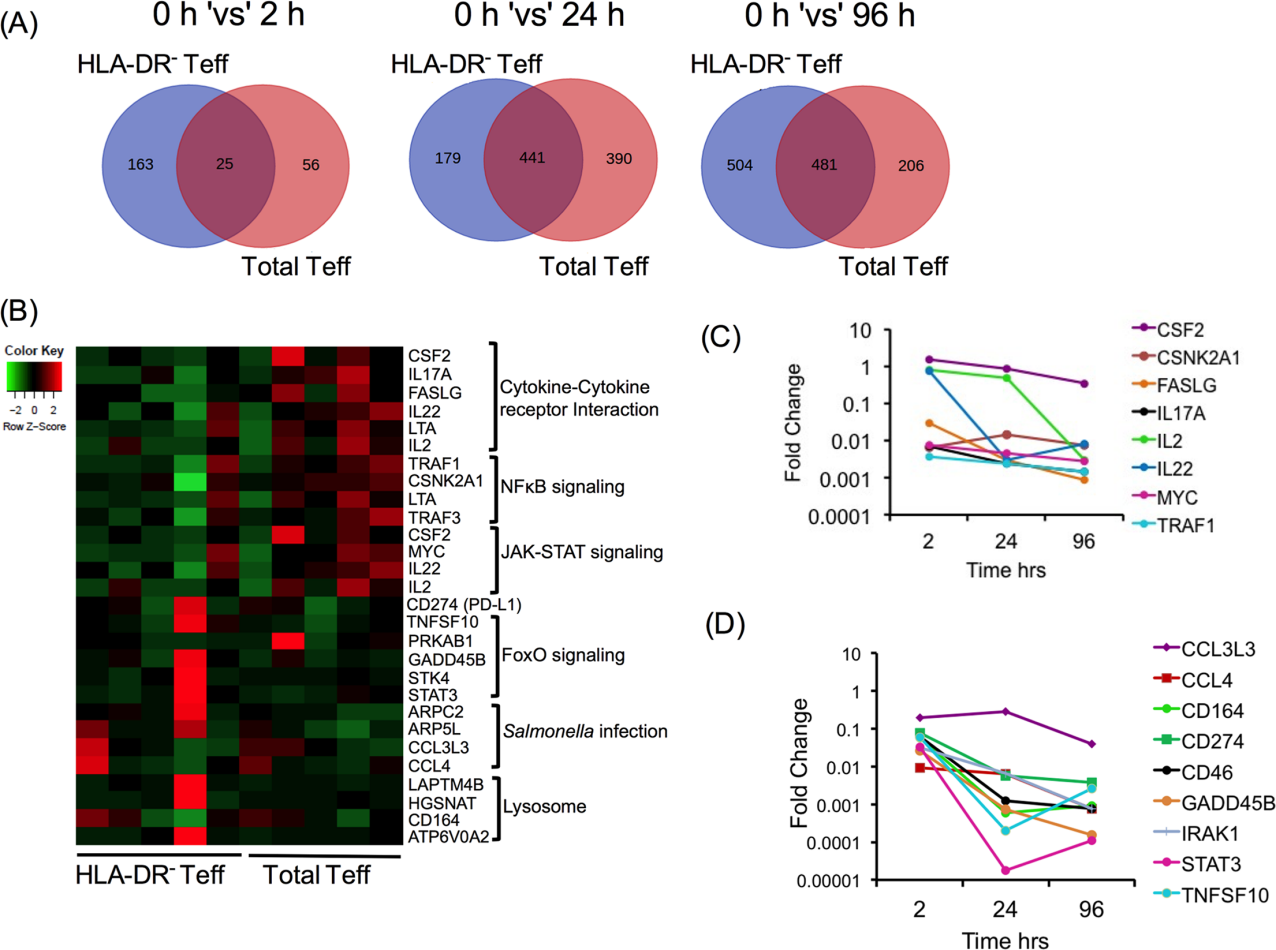
Transcriptome analysis reveals changes between total Teff and HLA-DR^-^ Teff cell subsets from PTB subjects. Total and HLA-DR^-^ Teff cells from PTB subjects were isolated by FACS and activated with anti-CD3/anti-CD28 activator beads (Teff:bead ratio of 1:1) for 2, 24 and 96 hrs. Unstimulated cells were used as 0 hr control. RNA was isolated at each time and subjected to RNA sequencing. A list of up-regulated DEGs (cut-off of log_2_ FC > 2.5, P < 0.05) was obtained by comparing gene expression post activation to that at 0 hr in both total and HLA-DR^-^ Teff cells. (A) Venn diagrams were drawn to identify unique and common genes up-regulated upon activation in the two cellular subsets of total and HLA-DR^-^ Teff cells. (B) Pathway analysis of unique genes over-expressed at 2 hrs post activation was done using DAVID Functional Annotation Bioinformatics Microarray Analysis tool. A heatmap was constructed using FPKM values of genes in enriched pathways using R software. As FPKM values are unevenly distributed, z score was calculated per row i.e. per gene for proper visualization. The colour key was adjusted accordingly. Mean fold change in expression of selected genes from enriched pathways at 2, 24 and 96 hrs post activation in total (C) and HLA-DR^-^ (D) Teff cells was plotted. A total of N = 5 at each time point for total and HLA-DR^-^ Teff cells was used for obtaining all data.

Total and HLA-DR^-^ Teff cells isolated by cell sorting from PTB subjects were cultured with anti-CD3/anti-CD28 mitogenic beads. RNA was isolated from these cultures prior-to and after 2, 24 and 96 hrs of stimulation and subjected to RNA-Sequencing analysis. The pattern of differentially expressed genes (DEG) was first determined by comparing changes in each population longitudinally over time after activation relative to baseline unstimulated control and then comparing DEG at each time point between the total and HLA-DR^-^ subsets. As anticipated all subjects displayed robust longitudinal DEG changes with time following T cell activation (S8 Fig). The pattern of DEGs, log_2_ fold change (log_2_ FC) ≥ 2.5, p ≤ 0.05, at each time point post activation relative to the unstimulated control is represented as a Venn diagram and the number of DEGs that are common and unique to each group listed (Fig 6A). A comprehensive list of genes is provided in S2 Table. Maximum changes in DEG number were observed at 96 hrs post activation in the HLA-DR^-^ fraction and at 24 hrs for the total Teff fraction (Fig 6A). In the HLA-DR^-^ Teff fraction the maximum number of unique DEGs observed was 504 at 96 hrs and in the total Teff cell fraction maximum unique DEGs were 390 at 24 hrs (Fig 6A). The percentages of unique genes in the HLA-DR^-^ Teff subset were 86.7%, 28.9% and 51.2% respectively at 2, 24 and 96 hrs post activation (Fig 6A). The number of unique genes in the total Teff population over time was: 69.1% at 2 hrs, 46.9% at 24 hrs and 29.9% at 96 hrs post activation (Fig 6A). We specifically mined the DEG list at 2 hrs post activation as this included the highest number of unique DEGs in HLA-DR^-^ Teff cells. Also, changes in gene expression early are a direct reflection of response to activation unlike at later time points, which may include genes impacted by those expressed early. A pathway analysis using DAVID of up-regulated DEGs at 2 hrs revealed genes involved in cytokine-cytokine receptor interaction (*CSF2*, *IL-17A*, *FASLG*, *IL-22*, *LTA*, *IL-2*), NF-кB signaling (*TRAF1*, *CSNK2A1*, *TRAF3*, *LTA*) and Jak-STAT signaling (*CSF2*, *MYC*, *IL-22*, *IL-2*) to be significantly up-regulated in total Teff compared to HLA-DR^-^ Teff cells (Fig 6B). On the other hand, FOXO signaling (*TNFSF10*, *PRKAB1*, *GADD45B*, *STK4*, *STAT3*), *Salmonella* infection (*ARPC2*, *ARPC5L*, *CCL3L3*, *CCL4*) and lysosome (*LAPTM4B*, *HGSNAT*, *CD164*, *ATP6V0A2*) pathways were up-regulated, albeit weakly, in the HLA-DR^-^ Teff subset (Fig 6B). Also, *CD274* or *PD-L1* was up-regulated in the HLA-DR^-^ Teff subset (Fig 6B). Time course analysis of DEG in these pathways showed expression of most of these genes to be highest at 2 hrs (Fig 6C and 6D), with expression of only some genes e.g. *CCL3L3*, *CCL4* (Fig 6C) and *CSF2* (Fig 6D) persistently elevated over time. In addition, CCL3 was also up-regulated in total Teff but its log_2_ FC was higher in HLA-DR^-^ Teff (log_2_ FC of 6.24 in HLA-DR^-^ ‘vs’ 4.00 in total Teff). This analysis therefore identified several candidates (IL-17, IL-22, CCL3L3, CCL4 and PD-L1), previously implicated in T cell function/regulation [33–38]. The expression of these genes was thus validated in the next set of experiments.

### HLA-DR^+^ Teff cells from PTB patients express higher levels of pro-inflammatory cytokines

**Fig 7:**
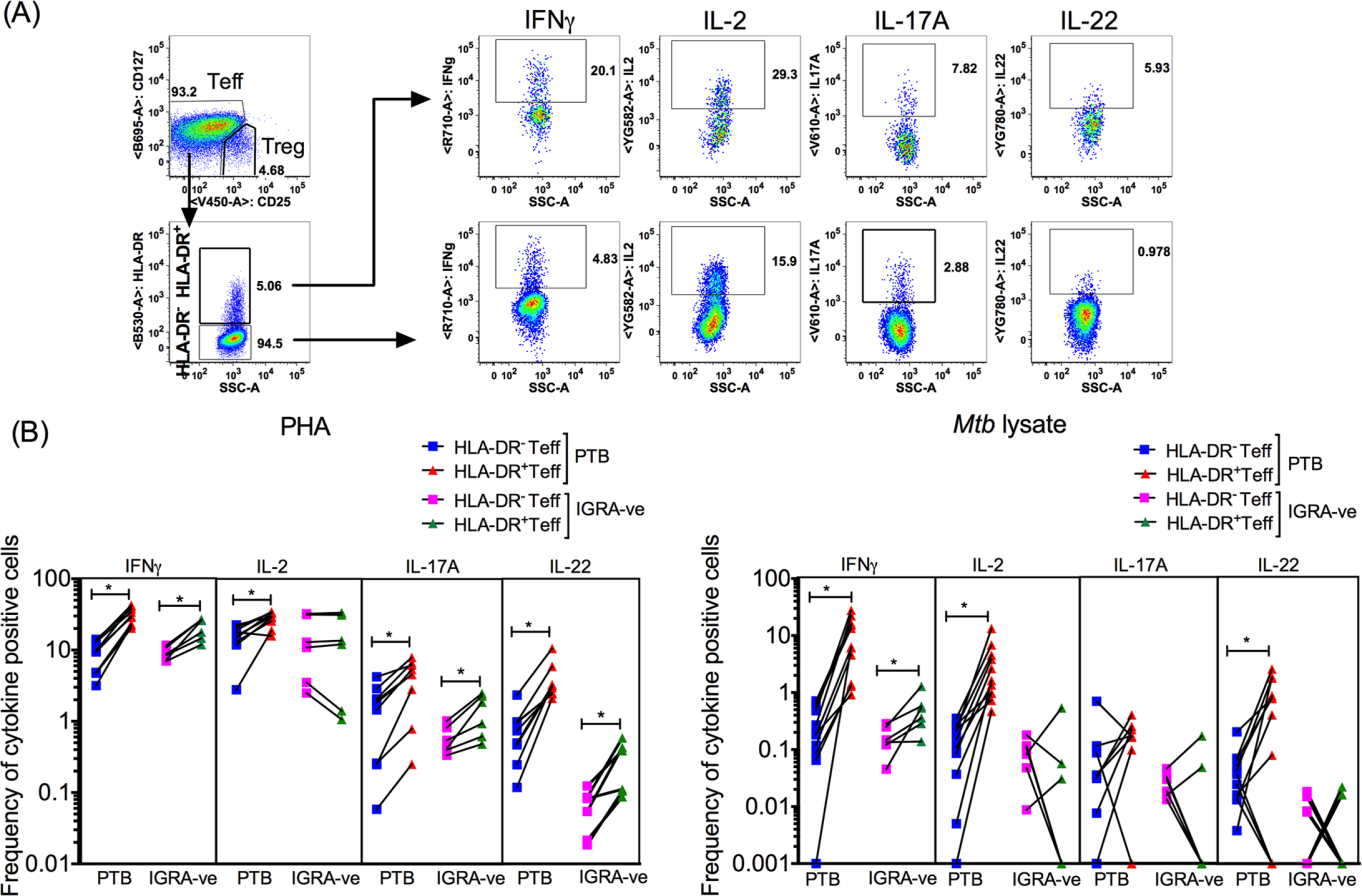
HLA-DR^+^ and HLA-DR^-^ Teff cells in PTB differ in their capacity to secrete pro-inflammatory cytokines. PBMC from PTB and IGRA-ve subjects were activated with either PHA or *Mtb* whole cell lysate. Brefeldin and monensin were added to cultures to prevent cytokine secretion. After 16 hrs of activation, cells were fixed, permeabilised and stained with an antibody cocktail comprising Avid, anti-CD3, anti-CD4, anti-CD45RA, anti-CD127, anti-CD25, anti-HLA-DR, anti-IFNγ anti-IL-2, anti-IL-17A and anti-IL-22. Expression of cytokines was measured in the Avid^-^CD3^+^CD4^+^CD45RA^-^CD127^hi^CD25^lo^HLA-DR^+^ and Avid^-^CD3^+^CD4^+^CD45RA^-^CD127^hi^CD25^lo^HLA-DR^-^ Teff compartments. A representative FACS plot of PHA activated PBMC from a PTB donor with cytokine positive cells in the HLA-DR^+^ and HLA-DR^-^ Teff fractions is shown (A). IFNγ^+^, IL-2^+^, IL-17A^+^ and IL-22^+^ cells in response to stimulation were measured as a frequency of HLA-DR^+^ and HLA-DR- Teff cells (B). A total of 9–11 PTB and 6 IGRA-ve donors were used. Paired Wilcoxon matched-pairs signed rank test with Bonferroni correction was used to determine *P* value. *p ≤ 0.013.

Prompted by the RNA sequencing data, we next studied if there were differences in expression of IL-2, IL-17A and IL-22 between HLA-DR^-^, HLA-DR^+^ and total Teff (includes HLA-DR^-^ and HLA-DR^+^ Teff) in the context of a wider panel of cytokines implicated in Teff function, including, IFNγ, and IL-10. PBMCs isolated from PTB patients were activated with either PHA or with whole *Mtb* lysate and cytokine expressing cell frequencies were measured by sequential gating within: Avid^-^CD3^+^CD4^+^CD45RA^-^CD127^hi^CD25^lo^ total Teff (includes both HLA-DR^+^ and HLA-DR^-^ cells), Avid^-^CD3^+^CD4^+^CD45RA^-^CD127^hi^CD25^lo^HLA-DR^-^ Teff cells and, Avid^-^CD3^+^CD4^+^CD45RA^-^CD127^hi^CD25^lo^HLA-DR^+^ Teff cells (Fig 7A, S10A Fig, for representative FACS plots). We found significantly higher frequencies of IFNγ^+^ cells in the HLA-DR^+^ Teff compartment compared to the HLA-DR^-^ Teff compartment under both stimulation conditions in PTB subjects (Fig 7B). In PTB subjects, IL-2 and IL-22 were also higher in HLA-DR^+^ compared to HLA-DR^-^ cells in response to both PHA and *Mtb* lysate stimulation (Fig 7B). Similar data was recorded for IL-17A in PHA stimulated cultures (Fig 7B), but not in response to *Mtb* antigen stimulation. This may be due to overall low frequency of *Mtb* specific IL-17A secreting cells reflected by the fact that several individuals were IL-17A non-responders (Fig 7B). There was no significant difference obtained in IL-10 expression between HLA-DR^+^ and HLA-DR^-^ Teff cells (S9 Fig). The increased IL-2, IFNγ and IL-22 expression in HLA-DR^+^ Teff in PTB was also reflected in higher expression of these cytokines in the mixed population of total Teff cells that contained HLA-DR^+^ and HLA-DR^-^ cells when compared to the HLA-DR^-^ Teff subset alone (S10B Fig). Thus, compared to their HLA-DR^-^ counterpart and consistent with their activated state, PTB HLA-DR^+^ Teff cells expressed 2–4 fold and 5–30 fold higher effector cytokines (IFNγ, IL-2, IL-17A and IL-22) in response to PHA and *Mtb* lysate respectively (Fig 7B).

To assess if elevated effector cytokine expression by HLA-DR^+^ cells was specific to PTB Teff (Fig 7B), a comparative analysis with cells from IGRA-ve controls was performed. Based on published data [21, 22, 45] which shows that HLA-DR^+^ T cells are activated cells, we predicted HLA-DR^+^ cells from IGRA-ve controls, like those of PTB, to include higher frequencies of cytokine expressing effectors compared to their HLA-DR^-^ counterpart. In confirmation, we show, IGRA-ve HLA-DR^+^ Teff cells expressed significantly higher IFNγ, IL-17A and IL-22 compared to HLA-DR^-^ Teff, particularly in response to polyclonal stimulation with PHA. However, the frequencies of *Mtb*-specific cytokine positive cells in IGRA-ve controls was low in keeping with ours [50] and previous studies [45, 51], showing higher frequencies of *Mtb*-specific CD4^+^ T cells in PTB, due to stimulation by ongoing infection and therefore, no significant difference was observed between HLA-DR^-^ and HLA-DR^+^ *Mtb*-specific Teff cells in IGRA-ve samples with the exception of the most abundant IFNγ^+^ *Mtb*-specific Teff cells (Fig 7B). A cross-sectional comparison of IGRA-ve ‘vs’ PTB HLA-DR^+^ IFNγ^+^/IL-2^+^/L-17A^+^/IL-22^+^ Teff confirms higher frequencies of these cells in PTB in response to both PHA and *Mtb* lysate stimulation (S11 Fig) consistent with overall higher frequencies of HLA-DR^+^ cells in PTB (Fig 4). These data confirm that the expanded population of HLA-DR^+^ Teff cells from PTB subjects express high levels of key pro-inflammatory cytokines—IFNγ, IL-2, IL-17A and IL-22 relative to HLA-DR^-^ Teff cells, thereby validating the RNA-Seq data. This phenomenon in turn has the potential to contribute to increased resistance of PTB Teff to Treg suppression through counter-regulation of Treg function.

### Blockade of cell surface CCR5 and PD-L1 reverses increased suppression observed in HLA-DR^-^ Teff cells from PTB patients

**Fig 8:**
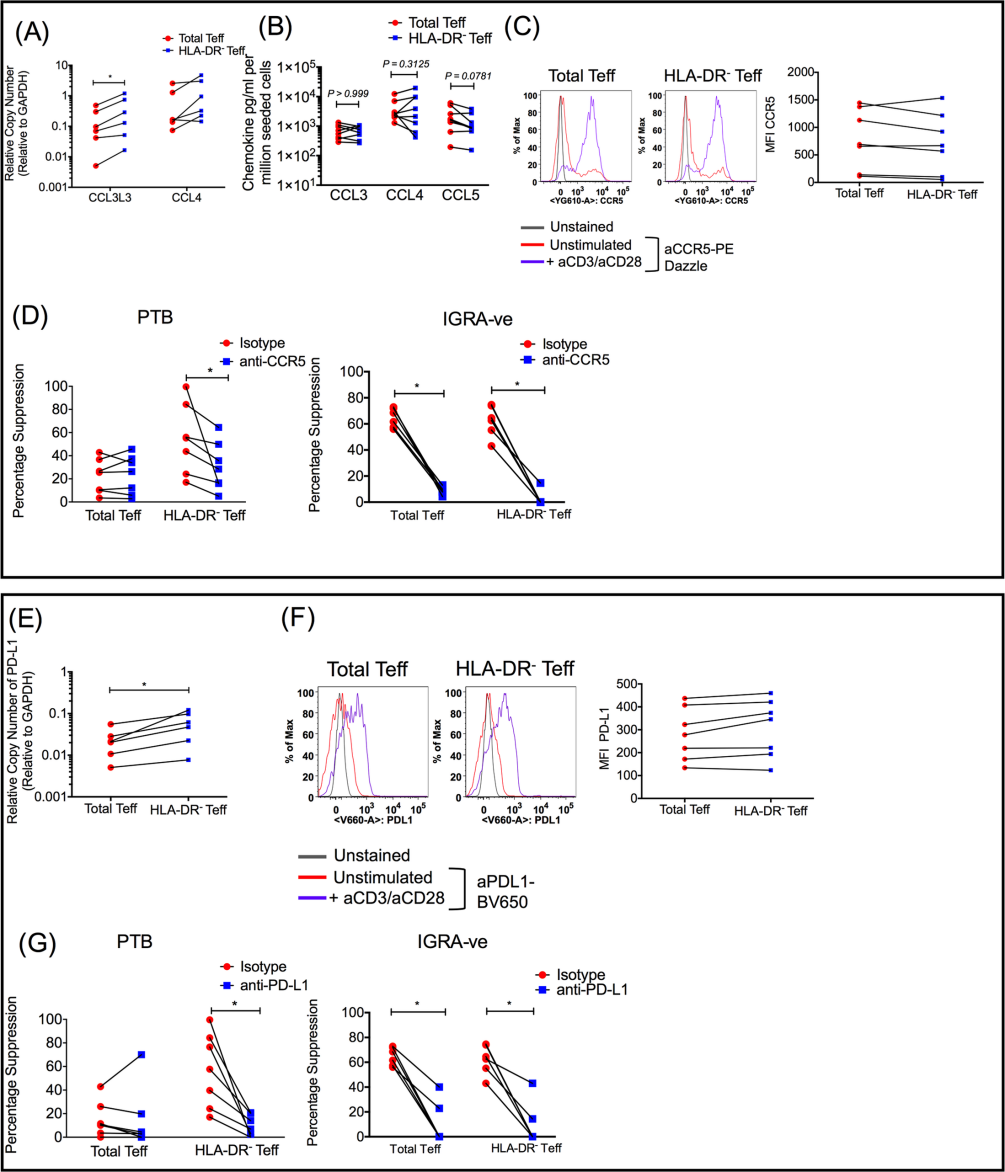
PTB HLA-DR^-^ but not HLA-DR^+^ total Teff are susceptible to Treg suppression via CCR5 and PD-L1. (A, E) RNA was isolated from sorted PTB total and HLA-DR^-^ Teff cells activated with anti-CD3/anti-CD28 for 2 hrs and converted to cDNA. Expression of *CCL3L3*, *CCL4* and *PD-L1* was measured by qRT-PCR. Expression is shown as relative copy number relative to GAPDH. (B) CCL3, CCL4 and CCL5 levels were measured in supernatants from cultures of PTB total and HLA-DR^-^ cells activated with anti-CD3/anti-CD28 for 24 hrs by ELISA. CCR5 (C) and PD-L1 (F) expression was measured on unstimulated and anti-CD3/anti-CD28 activated PTB total and HLA-DR^-^ Teff cells after 24 hrs and MFI of CCR5 (C) and PD-L1 (F) from multiple PTB donors was plotted for comparison between total and HLA-DR^-^ Teff cells. CFSE labelled sorted PTB and IGRA-ve total and HLA-DR^-^ Teff cells were co-cultured with autologous Treg cells at a ratio of 1:1 in the presence of either 5 μg/ml anti-CCR5 (D) or 5 μg/ml anti-PD-L1 (G). Appropriate 5 μg/ml isotype antibody was used as control in both cases and cells were activated with anti-CD3/anti-CD28 beads (beads:cell ratio of 1:1). Proliferation was measured by CFSE dilution after 4 days and percentage suppression was calculated (D and G). A total of 6–8 PTB and 6 IGRA-ve donors were used. Upper box shows data pertaining to CCR5 and lower box shows data pertaining to PD-L1. Paired Wilcoxon matched-pairs signed rank test with Bonferroni correction was used to determine *P* value. *p ≤ 0.03.

Next, we used qRT-PCR to validate DEGs implicated in T cell function that were enriched in the HLA-DR^-^ Teff subset from PTB patients. Specifically, we validated *CCL3L3*, *CCL4* and *PD-L1* (Fig 8A and 8E). qRT-PCR measurement showed higher expression of all three genes in HLA-DR^-^ Teff cells compared to total Teff cells, at 2 hrs post anti-CD3/anti-CD28 stimulation, with significance reached for *CCL3L3* (Fig 8A) and *PD-L1* (Fig 8E). However, protein levels of CCL3, CCL4 and CCL5 measured by ELISA at 24 hours post anti-CD3/anti-CD28 stimulation were not significantly different between PTB total and HLA-DR^-^ Teff cells (Fig 8B).

Previous studies have highlighted a potential role for chemokines CCL3 and CCL4 and their receptor CCR5 in Treg mediated suppression [33, 34]. To understand the importance of these pathways in Treg-mediated suppression in our system of study, we first measured expression of CCR5, the key receptor for beta chemokines: CCL3, CCL4 and CCL5, on PTB total and HLA-DR^-^ Teff at baseline and after activation (Fig 8C). CCR5 expression as measured by median fluorescence intensity (MFI) was similar on both subsets after activation (Fig 8C). Next, we investigated the effect of CCR5 blockade on Treg mediated suppression in activated PTB total and HLA-DR^-^ Teff cells (Fig 8D). Presence of anti-CCR5 antibody in an autologous suppression assay did not significantly alter the low level of suppression of PTB total Teff cells (Fig 8D). However, blockade of CCR5 in a suppression assay with PTB HLA-DR^-^ Teff cells significantly reduced Treg mediated suppression (Fig 8D) suggesting that: (a) the CCR5 pathway is important for suppression in PTB HLA-DR^-^ Teff cells, and (b) the presence of HLA-DR^+^ cells interferes with suppression mediated by this pathway, thereby contributing to the observed disparity in Treg mediated suppression of PTB total and HLA-DR^-^ Teff cells.

The functional significance of CD274 (PD-L1) was next tested (Fig 8E–8G). CD279 (PD-1) and CD274 (PD-L1) knockout mice show extreme sensitivity to *Mtb* infection [46]. Studies in humans show that PD-1^+^ and PD-L1^+^ CD4^+^ T cells to be higher in PTB [47] and their interaction leads to suppression of *Mtb* specific T cell responses [35, 36]. Further, blockade of the PD-1-PD-L1 interaction leads to inhibition of Treg function in cells harvested from PTB individuals [36]. These data are consistent with the importance of this pathway in both Teff and Treg function and therefore Treg-mediated homeostasis.

Although mRNA measurement showed PDL-1 to be marginally, but significantly higher on PTB HLA-DR^-^ compared to PTB total Teff (Fig 8E), cell surface staining showed similar expression at baseline and following activation (Fig 8F). However, the MFI of PD-L1 on PTB HLA-DR^-^ Teff cells was marginally higher (median MFI = 346, range = 123–461), compared to median MFI of PTB total Teff cells (median MFI = 278, range = 134–437) (Fig 8F) but there was no significant difference. Blocking with anti-PD-L1 was next carried out to test functional significance of PD-L1 expression. Blocking PD-L1 in autologous Treg suppression assays significantly diminished suppression of PTB HLA-DR^-^ Teff cells (Fig 8G) suggesting that enhanced suppression observed in HLA-DR^-^ Teff cells is partly mediated through PD-1/PD-L1 interactions. The reduction in percentage suppression in case of HLA-DR^-^ Teff cells upon blockade of PD-L1 was consistent across all donors tested and more marked than blocking the CCR5 pathway. Anti-PD-L1 decreased Treg suppression by 83% compared to a 43% drop in suppression of the same cells by anti-CCR5.

To assess if resistance of HLA-DR^+^ Teff to CCR5 and PDL-1-mediated suppression was specific to PTB, we analyzed the effect of blocking these pathways on suppression of Teff containing HLA-DR^+^ cells and the fraction depleted of this subset (HLA-DR^-^) from IGRA-ve controls. We predicted both these pathways to be operative based on data in Fig 4B, which showed that Teffs from IGRA- ve controls contain a very small percentage of HLA-DR^+^ cells and depletion of these cells did not affect Treg mediated suppression (Fig 5C). Furthermore, other data highlight both these pathways to be of fundamental importance for Treg suppression [33–37]. In support of this contention, we show that CCR5 and PDL-1 blockade significantly reduced autologous Treg mediated suppression of both total Teff containing HLA-DR^+^ cells and the fraction depleted of the HLA-DR^+^ subset in IGRA-ve control samples (Fig 8D and 8G). These data therefore highlight inherent functional differences between the expanded HLA-DR^+^ fraction in PTB ‘vs’ the minor subset of HLA-DR^+^ Teff in IGRA-ve subjects with both these pathways selectively compromised in the HLA-DR^+^ Teff fraction from PTB but not IGRA-ve subjects (Fig 8).

Taken together (Figs 7 and 8), these data confirm that resistance of HLA-DR^+^ Teff cells from PTB subjects to Treg mediated suppression is potentially a combination of expression of high levels of Th17 cytokines that can block Treg function and reduced sensitivity to CCR5 and PD-L1 mediated suppression by Treg cells.

### NFκB activation through CCR5 and PD-L1 ligation is impaired in HLA-DR^+^ containing Teff cells from PTB patients

**Fig 9:**
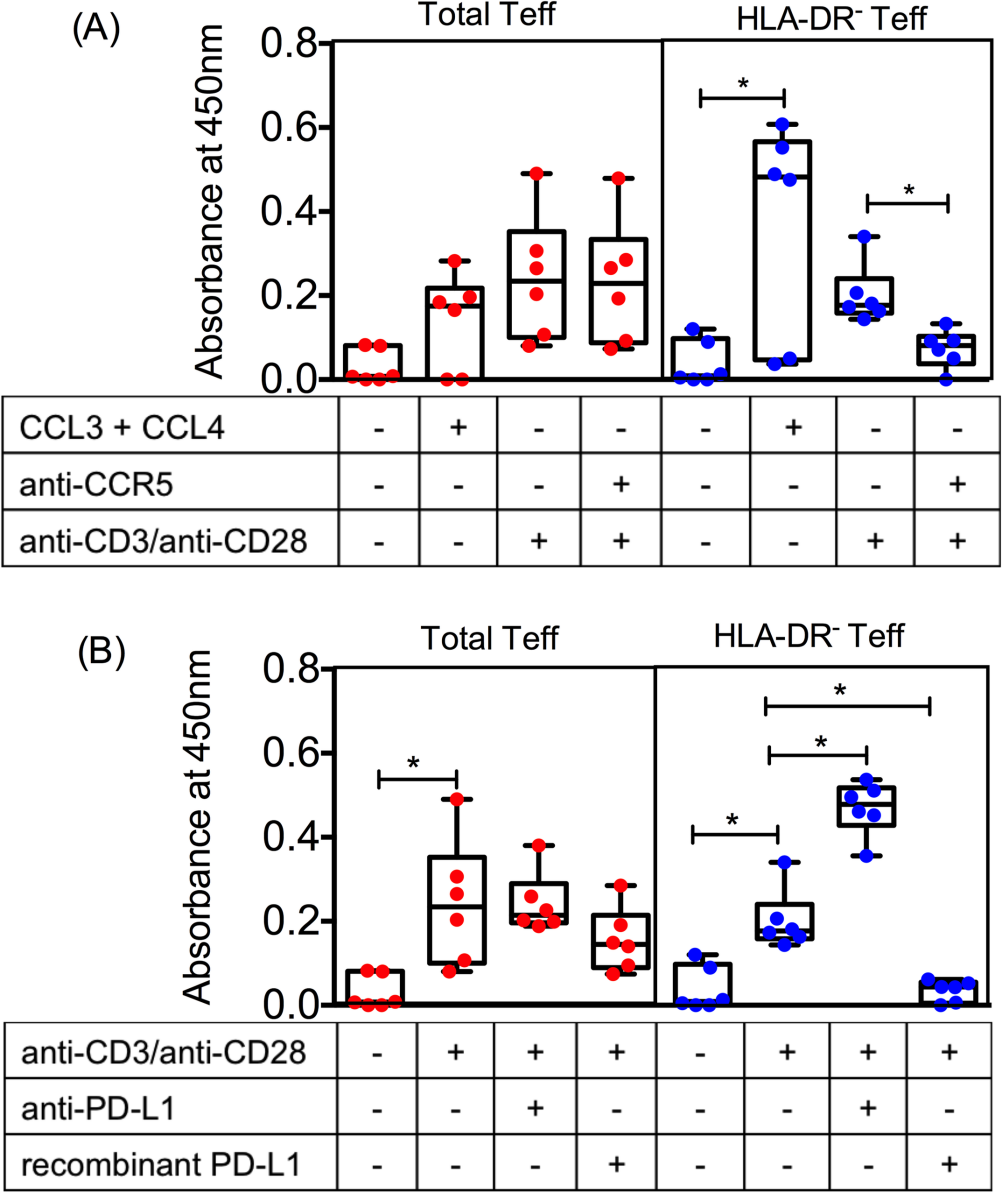
PTB Teff containing HLA-DR^+^ subset are compromised in CCR5 and PD-L1 mediated signalling. Sorted PTB total and HLA-DR^-^ Teff cells were treated with 50 ng/ml each of CCL3 and CCL4 (A), 5 μg/ml anti-CCR5 (A), 5 μg/ml anti-PD-L1 (B) or 10 μg/ml recombinant PD-L1 (B). Cells were activated with anti-CD3/anti-CD28 beads (beads:cell ratio of 1:1). Anti-CCR5, anti-PD-L1 and recombinant PD-L1 were added 20 minutes prior to addition of mitogenic anti-CD3/anti-CD28. Unstimulated cells were used as control. NFκB activation was measured by ELISA after 180 minutes of stimulation and was expressed as absorbance at 450 nm. (A) shows data pertaining to CCR5 mediated signaling and (B) shows data pertaining to PD-L1 mediated signaling. Data shows median and maximum and minimum values from N = 6 PTB donors. Paired Wilcoxon matched-pairs signed rank test with Bonferroni correction was used to determine *P* value. *p ≤ 0.03.

We next explored if reduced sensitivity of HLA-DR^+^ containing Teff cells to CCR5 and PDL-1 mediated suppression was due to impaired signaling via these pathways using total and HLA-DR^-^ purified Teff subsets from PTB patients. We studied effects of addition of ligands CCL3/CCL4 and recombinant PD-L1 and blocking antibodies anti-PD-L1 and anti-CCR5 on NFκB activation (Fig 9). Binding of ligands CCL3 and CCL4 to their receptor CCR5 triggers NFκB activation [52]. Similarly, PD-1 and PD-L1 interaction dampens proximal TCR signaling events including NFκB activation [53]. NFκB activation was significantly elevated upon addition of CCR5 ligands CCL3 and CCL4 only in the HLA-DR^-^ Teff subset (Fig 9A). Similarly, blockade of CCR5 resulted in significant dampening of anti-CD3/anti-CD28 mediated NFκB activation only in the HLA-DR^-^ Teff subset (Fig 9A). Further, blockade of PD-L1 resulted in significant increase in anti-CD3/anti-CD28 mediated NFκB activation only in the HLA-DR^-^ Teff subset (Fig 9B). Similarly, addition of recombinant PD-L1 significantly dampened anti-CD3/anti-CD28 mediated NFκB activation only in the HLA-DR^-^ Teff subset (Fig 9B). Again to test specificity, we performed similar experiments in total and HLA-DR^-^ Teff cells from IGRA-ve donors (S12 Fig). We found that addition of CCL3 + CCL4 increased NFκB activation in both total and HLA-DR^-^ Teff cells (S12A Fig). Similarly, addition of anti-CCR5 reduced NFκB activation in both total and HLA-DR^-^ Teff cells (S12A Fig). Addition of anti-PD-L1 elevated NFκB activation consistent with the blocking of a negative co-stimulatory signal and concomitantly, addition of recombinant PD-L1 dampened NFκB activation in both total and HLA-DR^-^ Teff IGRA-ve cells (S12B Fig). Taken together, data in Fig 9 and S12 Fig confirm selective loss of CCR5 and PD-L1 signaling in T effectors containing HLA-DR^+^ Teff cells from PTB but not IGRA-ve subjects.

## Discussion

Data presented in this study provides fresh insight into dysregulation of Treg mediated homeostasis and the possible mechanisms underpinning this in active PTB. Novel aspects of the study are (a) loss of Treg-mediated function in TB is due to Teff cells becoming resistant to Treg mediated suppression rather than inability of Treg cells to suppress Teff cells or a drop in circulating Treg cell frequency (b) this resistance is due to expansion of a small population of HLA-DR^+^ CD4^+^ Teff cells as depletion of this fraction restores suppression to control levels, and (c) that a combination of mechanisms accounts for the resistance of total HLA-DR^+^ Teff cells to suppression by Treg cells, which includes expression of high levels of IFNγ, IL-17A and IL-22, known to counter-regulate Treg cell function [38, 39, 54]. Additionally, HLA-DR^+^CD4^+^ Teff cells possibly interfere with PD-L1 and CCR5 mediated Treg-suppression, as depleting the HLA-DR^+^ fraction restores sensitivity of Teff cells to suppression via these pathways.

Tregs possibly play opposing roles in TB. A detrimental role, early post infection, is based on murine models showing an inappropriate accumulation of pathogen specific thymus derived Tregs following transportation of *Mtb* to the pulmonary lymph node after aerosol exposure, which inhibits and thereby delays induction of a protective T cell response resulting in higher bacterial burden [10, 55]. On the other hand, a potentially protective role for Treg cells in the chronic phase, linked to suppression of inflammation, is based on both murine and macaque models. Absence of Tregs in lungs of infected mice can lead to uncontrolled inflammation and mortality [28]. Also, depletion of Tregs from infected mice does not decrease pathogen load [56]; rather their expansion with IL-2 therapy provides resistance to TB in infected macaques [29]. Our data is consistent with a potentially beneficial role for Treg-mediated regulation in the chronic phase of TB by limiting the damaging effects of chronic T cell activation.

Variable changes in circulating Treg frequencies have been reported across different TB cohorts [12–18]. We also found a wide range of Treg frequency in PTB with no significant difference compared to controls. This inherent diversity in circulating Treg frequencies may reflect the efficiency by which Tregs traffic and home to the site of infection in the lung, where Treg cells have been previously noted. Studies in humans found that Tregs expand more strongly at local sites of infection than systemically [12, 57]. Beyond measure of frequency, several studies show that Treg cells isolated from human subjects with PTB preserve their suppressive potential [13, 14, 36, 58]. We confirm this to be the case, but additionally highlight that despite maintaining suppressive potential, Treg-mediated function is compromised in PTB subjects due to Teff cell resistance to Treg suppression. Whilst such resistance has not been reported in PTB, it is consistent with data from autoimmune conditions where activated T cells contribute to pathology and become resistant to Treg mediated suppression [32, 43, 59–61]. Consistent with our data, deleterious T cell activation in TB has been linked to expansion of a minor subset (median frequency = 9.5%, Range = 4.12–40%) of HLA-DR^+^ and or CD38^+^ T cells [21, 22, 45]. In latent and active TB subjects co-infected with HIV, the frequency of activated CD38^+^, HLA-DR^+^ T cells is elevated [25], and *Mtb* specific IFNγ^+^Ki67^+^ cell frequency co-expressing HLA-DR and CD38 is reportedly higher in PTB compared to latent TB subjects [45]. Importantly, elevated frequencies of CD4^+^HLA-DR^+^ cells have been identified to be a potential predictive marker of active TB risk in both BCG vaccinated infants and *Mtb* infected adolescents [21].

The precise mechanisms underpinning how CD4^+^HLA-DR^+^ T cells might contribute to TB disease have not been elucidated. We demonstrate for the first time through depletion studies that it is the small fraction of HLA-DR^+^, rather than CD38^+^ or PD-1^+^ Teff cells, that impairs Treg-mediated homeostatic control and we used differential transcriptome analysis of HLA-DR^+^ total Teff ‘vs’ effectors depleted of HLA-DR^+^ cells to identify genes that contribute to this observation. One key mechanism is through significantly elevated expression of key Th1 effector cytokine, IFNγ, and Th17 cytokines IL-17A and IL-22. Th17 cells though important in immunity to infection also contribute to damaging inflammation, if uncontrolled [62]. IL-22 is a member of the IL-10 cytokine family [63] and can be pathological when co-expressed with IL-17 and IFNγ [63–65]. Importantly, Th17 and Treg subsets are counter-regulatory. The function of Treg cells is recognised to be influenced by IFNγ [39] and their generation is reciprocally regulated along with Th17 differentiation by IL-6 and TGFβ [38]. Indeed, a high Th17/Treg ratio is linked to disease in rheumatoid arthritis [66]. The Th17/Treg ratio has also been reported to be altered in subjects with TB relative to healthy controls [67–70]. Our data provides mechanistic insight by identifying elevated Th17 cytokine expression by HLA-DR^+^ Teff as a potential mechanism for their resistance to Treg-mediated suppression.

Our RNA-Sequence data combined with blocking studies highlight that HLA-DR^+^ cells can additionally impair Treg-mediated regulation through CCR5 and PD-L1 pathways. A role for CCL3, CCL4, CCL5 and their receptor CCR5 in Treg mediated suppression has been previously reported in autoimmunity and cancer [33, 34] and we demonstrate through blockade experiments both these pathways to be of fundamental importance in cells from IGRA-ve controls. Expression of beta-chemokines by Tregs and CCR5 on Teff promotes suppression through migration of Treg cells [33, 34]. CCR5 can also function as a T cell co-stimulator [71]. In addition, CCR5 signaling has been implicated in the resolution of inflammatory responses through IL-10 expression [72–74]. On the other hand, PD-L1, a ligand for a negative co-stimulatory receptor PD-1, is expressed on antigen presenting cells as well as on activated T cells [75]. PD-1-PD-L1 interaction inhibits T cell activation and several effector T cell responses and helps in maintaining immune tolerance [76, 77]. In addition, PD-1-PD-L1 interactions have been reported to increase Treg generation and function by enhancing FoxP3 and sustaining its expression [37]. Elevated frequency of PD-L1^+^ CD4^+^ T cells has been reported in PTB subjects [47] with blocking studies highlighting this pathway to be functional [36]. We found that HLA-DR^+^ Teff resistance to Treg suppression mediated through the CCR5 and PD-L1 pathways is not due to absence of CCR5 or PD-L1. Instead, our data demonstrate that NFkB signalling via both CCR5 and PD-L1 ligation on PTB Teff cells is selectively impaired in HLA-DR^+^CD4^+^ T cells reflected by antibody mediated blockade of these pathways rescuing Treg-mediated suppression in the fraction of Teff cells depleted of HLA-DR^+^ cells but not the Teff fraction containing HLA-DR^+^ cells from PTB subjects. Comparative analysis with Teff from IGRA-ve controls, highlights fundamental functional differences between HLA-DR^+^ Teff cells from PTB ‘vs’ IGRA-ve subjects as the CCR5 and PD-L1 pathways remained functional in the fraction of Teff containing HLA-DR^+^ cells isolated from IGRA-ve but not PTB subjects. One mechanism for this functional difference may be related to lower expression of counter-regulatory cytokines (IFNγ, IL-22 and IL-17A) by IGRA-ve compared to PTB HLA-DR^+^ cells reflected also by significantly lower overall frequencies of memory HLA-DR^+^CD4^+^ Teff cells in IGRA-ve ‘vs’ PTB. These data raise the important possibility that activated memory CD4^+^ T cells identified by HLA-DR expression may be inherently functionally different across different disease states, which is a testable hypothesis and subject of future investigation.

Beyond the above tested pathways, our RNA-sequence data highlights that Teff cells comprising the HLA-DR^+^ fraction express higher levels of markers of inflammation and T activation (*CSF2*, *FASLG*, *TRAF1* and *TRAF3*) [78–81]. On the other hand, HLA-DR^-^ Teff cells, apart from *PD-L1*, *CCL3L3* and *CCL4*, had elevated expression of complement receptor *CD46* and *TRAIL*. CD46 is important for Treg activation and CD46 or 'complement activated' Tregs can suppress *Mtb* specific CD4^+^ T cell responses [82]. TRAIL promotes Treg proliferation and inhibits Th1 responses, consequently suppressing autoimmunity [83]. Therefore, total HLA-DR^+^ Teff cells have a molecular signature associated with enhanced inflammation and activation while HLA-DR^-^ Teff cells have a signature comprising molecules involved in regulation and immune homeostasis.

It is well recognised that PTB subjects have multiple immune differences to that of healthy controls. Gene signatures that distinguish PTB from healthy controls have been clearly identified by both large-scale biomarker studies [19, 84–86], as well as focussed studies that have probed the importance of individual genes/pathways that differ between PTB and controls [87–89]. The current study was designed to provide mechanistic insight to disease process in the context of a novel phenomenon: namely, resistance of CD4^+^ memory T effectors from PTB subjects to Treg-mediated suppression. Whilst other pathways may be operative, we provide unequivocal data to show that circulating HLA-DR^+^CD4^+^ effector memory T cells resistant to CCR5 and PD-L1 mediated suppression compromise regulatory T cell function in tuberculosis. These data provide fresh insight to how immune activation through expansion of HLA-DR^+^CD4^+^ memory T cells may contribute to disease in TB and is consistent with the emerging paradigm that TB pathology, like other chronic infections, is associated with T cell immune activation [19, 20, 88, 90, 91]. Our data identifies HLA-DR^+^ Teff cells resistant to Treg suppression as a potential functional marker of disease with implications for disease monitoring and response to therapy.

## Materials and methods

### Ethics statement

This study was conducted according to the principles expressed in the Declaration of Helsinki. Ethical approval was obtained from the Ethical Review Committee of St. John’s Medical College Hospital, Bangalore, India (Ref no: 55/2015). All study participants were adults and provided written informed consent for the collection of samples and subsequent analyses.

### Clinical subjects

Individuals were prospectively recruited between April 2015 and November 2017. Blood was collected by venepuncture in ACD (BD, Franklin Lakes NJ, USA) or EDTA (BD, Plymouth, UK) Vacutainer tubes. Matched sputum samples were also collected for individuals suffering from pulmonary tuberculosis (PTB). Clinical information of study participants are summarized in Table 1 and described in detail in S1 Table. Four clinical groups were studied as described below:

(1) IGRA+ve and IGRA-ve

Healthcare workers of St. John’s National Academy of Health Sciences and staff at Centre for Infectious Disease Research, Indian Institute of Science, Bangalore were recruited through internal calls highlighting the nature and importance of the study. Study subjects were classified as IGRA+ve based on results of a standard QuantiFERON TB Gold In-tube IGRA test (Qiagen, Germany) which measures IFNγ release in response to TB antigens. IFNγ release in response to TB antigens for IGRA+ve subjects was an average of 3.055 IU/ml (range = 0.36 - >10) and for IGRA-ve subjects was 0.02 IU/ml (range = 0–0.18). A total of 16 IGRA-ve, median age 28.5 years and 14 IGRA+ve, median age 32.5 years were included in the study (Table 1, S1 Table). All enlisted IGRA+ve individuals had not received preventive/curative therapy for TB in the past.

(2) Pulmonary TB (PTB)

A total of 45 donors with a median age of 37 years were included in the study (Table 1). Donors for this prospective study were enrolled from the Revised National Tuberculosis Program (RNTCP) clinic of St. John’s Medical College and Hospital. A diagnosis of PTB was ascertained by sputum smear microscopy and culture. Standard smear grading of 1^+^, 2^+^ and 3^+^ was used to ascertain the bacterial burden. In several cases a chest X-ray was performed, and sputum samples were collected for reconfirmation of TB diagnosis by GeneXpert MTB/RIF (presence of *Mtb* bacilli and rifampicin resistance) assay (Cephid, USA). All subjects were treatment naive at the time of enrolment. All donors included in the study were smear positive; 22 had abnormal chest X-ray; 38 were positive and 3 negative for *Mtb* by GeneXpert; 35 were negative and 3 were positive for RIF resistance by GeneXpert (Table 1, S1 Table).

(3) Pulmonary TB treated

Study subjects were counselled and initiated on standard anti-TB treatment (ATT) based on the directly observed treatment, short course (DOTS) regimen. Samples from 9 cross-sectional donors having undergone ATT, median age 30, were used in the study (Table 1). At a follow up visit 6 month from the start of ATT, blood was collected in EDTA or ACD tubes for isolation of PBMC. Three donors were positive and 6 were negative for presence of *Mtb* bacilli as determined by GeneXpert. One donor had RIF resistance (Table 1, S1 Table).

### Isolation of PBMC from blood

Peripheral blood (30 ml) was collected by venepuncture in (BD, Franklin Lakes NJ, USA) or EDTA (BD, Plymouth, UK) Vacutainer tubes. It was diluted 2-fold with PBS (Gibco by Life Technologies, Washington, DC, USA) + 2% FBS (Gibco by Life Technologies, Washington, DC, USA), layered onto an equal volume of Ficol Histopaque (Sigma Aldrich, St. Louis, MO, USA) and centrifuged at 200g for 20 minutes. Interface containing PBMC was carefully removed and cells were washed twice with PBS + 2% FBS and frozen in cryopreservation solution comprising 10% DMSO (Sigma Aldrich, St. Louis, MO, USA) and 90% FBS (Gibco by Life Technologies, Washington, DC, USA) in liquid nitrogen till future use.

### Isolation of effector and regulatory CD4^+^ T cells using magnetic beads

Vials of frozen peripheral blood mononuclear cells (PBMC) were taken out in a liquid nitrogen bath. They were thawed in a 37°C water bath till a pea sized lump of frozen material remained in the vial. One ml of pre-warmed RPMI (Gibco by Life Technologies, Washington, DC, USA) + 10% FBS + antibiotics (Gibco by Life Technologies, Washington, DC, USA) was added to the vials and the total contents were transferred drop–by–drop to a 50 ml tube containing 20 ml of warm complete media. Cells were pelleted down at 800g for 5 min and then subjected to another wash with complete media. Cells were re-suspended in complete media and rested in 6 well plates at a density of 4–5 x 10^6^/ml. Cells were rested for 1–2 hrs after which they were washed, counted and 10–20 x 10^6^ cells were used for isolating subsets of CD4^+^CD25^-^ Teff and CD4^+^CD25^+^ Treg cells with a Dynabeads human Treg isolation kit (Invitrogen by Life Technologies, Oslo, Norway) following the manufacturer’s instructions. Briefly, cells were incubated with 0.5μg/10 x 10^6^ cells of anti-CD45RA (clone HI100, eBiosciences, San Diego, CA, USA) along with a depletion antibody cocktail followed by magnetic depletion beads to negatively isolate CD45RA^-^CD4^+^ memory Teff cells. These cells were next used to positively isolate CD45RA^-^CD4^+^CD25^+^ Treg cells. The cells remaining after positive isolation of Treg cells were further depleted of CD4^+^CD25^+^ cells to obtain CD45RA^-^CD4^+^CD25^-^ effector cells. Purity of cells was assessed by staining with anti-CD4, anti-CD3 and anti-CD25. The percentage of contaminating CD25^+^ Treg cells in the CD25^-^ Teff population was < 10% and the purity of CD25^+^ Treg cells was ≥ 90%.

### Effector and regulatory CD4^+^ T cell sorting

Frozen PBMC were thawed as previously described. They were rested and then washed and resuspended in cold PBS + 2% FBS. Live/dead fixable Aqua dead cell stain or Avid (Molecular Probes, Life Technologies, Oregon, USA) was added to the cells at a concentration of 0.125 μl/100 μl of staining volume for a period of 10 min at 4°C. 5 ml of PBS + 2% FBS was added to the cells and an aliquot of 15–20 μl was removed at this stage to serve as Avid compensation control. Remaining cells were pelleted at 800g for 5 min. A staining cocktail of total 100 μl volume comprising anti- CD3 BV570 (Clone UCHT1, Biolegend, San Diego, CA, USA), anti-CD4 BUV395 (Clone SK3, BD Biosciences, USA), anti-CD45RA APC-H7 (Clone HI100, BD Biosciences, USA), anti-CD127 PerCP-Cy5.5 (Clone eBioRD5, eBiosciences, San Diego, CA, USA), anti-CD25-PE (Clone 2A3, BD Biosciences, USA) and anti-CD25-PE (Clone MA251, BD Biosciences, USA) was prepared in PBS + 2% FBS. For depletion of CD38^+^, HLA-DR^+^ and PD-1^+^ Teff cells the staining cocktail was modified to include anti-CD25 BV421 (Clone MA251, BD Biosciences, USA), anti- HLA-DR FITC (Clone G46-6, BD Biosciences, USA), anti-PD-1 PE-Cy7 (Clone eBioJI05, eBiosciences, San Diego, CA, USA) and anti-CD38 PE (Clone HIT2, BD Biosciences, USA). Cells were incubated with the staining cocktail for 10 min at 4°C. Finally, cells were washed in cold PBS + 2% FBS + 25 mM HEPES (Sigma Aldrich, St. Louis, MO, USA), resuspended in the same buffer (total volume– 200 μl) and added to 5 ml tubes with cell strainer caps (Falcon, Corning, Mexico) to remove clumps. Sorting was carried out using a BD FACS Aria Fusion with a 100 μm nozzle, 15 psi, 100 rpm agitation at 4°C with a flow rate of 3000–6000 events/sec. The 4-way purity parameter was selected for maximum purity. Sorted Teff cells were collected in 1 ml RPMI+10% FBS and Treg cells were collected in 200 μl FBS in 5ml polypropelene FACS tubes (Sarstedt, Numbrecht, Germany). The tubes were briefly coated with FBS before use to minimise cell adherence to plastic. Appropriate single colour stained, and unstained samples were used as compensation controls. CD3^+^CD4^+^CD45RA^-^CD127^hi^CD25^lo^ cells were sorted as memory Teff and CD3^+^CD4^+^CD45RA^-^CD127^lo^CD25^hi^ were sorted as Treg and used in supression assays. Additionally, for some experiments, Teff depleted of CD38^+^, HLA-DR^+^ and PD-1^+^ cells were obtained. In some cases, Tregs and PBMC minus Treg (comprising all PBMC depleted of Tregs) fractions were sorted. Purity of sorted cells was gauged by performing a post-sort analysis. Purity of sorted populations was close to 100%.

### Treg suppression assay

Purified Treg and Teff cells were used for suppression assay. Teff cells were resuspended in 1 ml warm PBS + 1% FBS and Tregs in 1ml RPMI + 10% FBS + antibiotics. Cells were counted and Teff were labelled with CFSE as follows: to each 1 ml of Teff cells, 2 μl of 5mM CFSE (Molecular Probes, Life Technologies, Oregon, USA) was added and incubated at 37°C for 7 minutes in the dark, after which CFSE uptake was stopped by addition of 5 ml ice-cold complete RPMI media. Cells were pelleted down washed once with complete RPMI and used in a suppression assay. Teff cells were seeded at a density of 5000/well. Treg cells were added at different ratios to the Teff cultures. Washed anti-CD3/anti-CD28 T cell activator beads (Gibco by Thermofisher Scientific, Vilnius, Lithuania) were added at beads:Teff ratio of 1:1 for sorted Teff cells and 2:1 for Teff isolated by magnetic beads. In some cases, heterologous cross-over assays were set up where Teff and Treg cells from different individuals were co-cultured. Also, under some conditions suppression assays were set up in presence of either 5 μg/ml anti-CCR5 (mouse monoclonal, clone 45531, R & D Systems, MN, USA), 5 μg/ml anti-PD-L1 (Clone 29E.2A3, Biolegend, San Diego, CA, USA) or 5 μg/ml isotype control mouse IgG2b (monoclonal, clone 20116, R & D systems, MN, USA). CFSE dilution was measured after 4 days of culture using BD FACS CantoII or BD FACSVerse. Percentage suppression was calculated using the formula: (Avg proliferation without suppressors–proliferation with suppressors)/Avg proliferation without suppressors x 100. For some experiments, cells were activated with 10 μg/ml *Mtb* H37Rv lysate (BEI Resources, Manassas, VA, USA) instead of anti-CD3/anti-CD28 beads. In some experiments IFNγ secretion as measured by ELISA was used instead of proliferation as a functional readout. Percentage suppression in such instances was calculated using the formula: IFNγ pg/ml secreted per 10^6^ cells without suppressors–IFNγ pg/ml secreted per 10^6^ cells with suppressors)/ IFNγ pg/ml secreted per 10^6^ cells without suppressors x 100.

### Flow cytometry for estimation of Treg frequency and expression of other cell surface markers

Frozen peripheral blood mononuclear cells (PBMCs) were thawed and rested for 2 hrs in RPMI + 10% FBS at 37°C. Cells were washed, counted and 1 x 10^6^ were used for staining. For estimation of Treg frequencies cells were first stained with a cocktail of comprising pre-titrated viability dye Avid (Molecular Probes, Life Technologies, Oregon, USA), anti-CD3 BV570 (clone UCHT1, Biolegend, San Diego, CA, USA), anti-CD4 BUV395 (clone SK3, BD Biosciences, USA), anti-CD25 PE (Clone 2A3, BD Biosciences, USA) + anti-CD25 PE (clone MA251, BD Biosciences, USA) or anti-CD25 BV421 (clone MA251, BD Biosciences, USA), anti-CD45RA APC-H7 (clone HI100, BD Biosciences, USA) and anti-CD127 PerCP-Cy5.5 (clone eBioRD5, eBiosciences, San Diego, CA, USA). Next, cells were fixed and permeabilized using the FoxP3 staining buffer set (eBiosciences, San Diego, CA, USA) according to manufacturer’s instructions and stained with anti-FoxP3 Alexa fluor 647 (clone 259D/C7, BD Biosciences, USA). For characterisation of Teff cells the extracellular staining antibody cocktail was modified to include anti-HLA-DR FITC (clone G46-6, BD Biosciences, USA), anti-CD38 PE (clone HIT2, BD Biosciences, USA) and anti-PD-1 PE-Cy7 (clone eBioJI05, eBiosciences, San Diego, CA, USA). For some experiments cells were also stained with anti-CCR5 PE Dazzle (cloneJ418F1, Biolegend, San Diego, CA, USA) and anti-PD-L1 BV605 (clone MIH1, BD Biosciences, USA). At the end of all staining procedures, cells were washed and fixed with 1% paraformaldehyde (Electron Microscopy Sciences, Hatfield, PA, USA) and analysed on a BD FACSVerse or BD ARIA Fusion flow cytometers. Individually stained beads (eBiosciences, San Diego, CA, USA) were used as compensation controls. Data was analysed using FlowJo software (FlowJo LLC, Ashland, Oregon, USA).

### ELISA for CCL3, CCL4, CCL5 and IFNγ

Supernatants from cultures were collected at specified times. Chemokines CCL5, CCL4 and CCL3 were measured in the culture supernatants using ELISA kits (R & D Systems) according to manufacturer’s instructions. A standard sandwich ELISA was performed in each case using a separate coating and detection antibody. Bound chemokine was detected finally by the enzymatic conversion of o-phenylenediamine dihydrochloride or OPD (Sigma) by HRP conjugated to the secondary antibody. Colour was read at 492 nm using VERSA Max Microplate Reader (Molecular Devices). Absolute concentration of chemokine was calculated using a standard curve. The linear range of detection for CCL5 was 18.75–2400 pg/ml, for CCL4 was 18.75–1200 pg/ml and for CCL3 it was 18.75–600 pg/ml. For IFNγ, ELISA was performed according to manufacturer’s instructions (BD Biosciences, USA). Absolute concentration of IFNγ was calculated using a standard curve with a linear range of detection of 10–1250 pg/ml.

### ELISA for activated NFkB

NFkB ELISA was performed using a commercially available kit following manufacturer’s instructions (Active Motif, Belgium). Briefly, 105 sorted cells were treated with 50 ng/ml of CCL3/CCL4 (R & D systems, MN, USA), 5 μg/ml anti-CCR5 (mouse monoclonal, clone 45531, R & D Systems, MN, USA), 5 μg/ml anti-PD-L1 (Clone 29E.2A3, Biolegend, San Diego, CA, USA) or 10 μg/ml recombinant PD-L1 (R & D systems, MN, USA). Cells were stimulated with anti-CD3/anti-CD28. Unstimulated cells were used as controls. Cells were lysed after 180 minutes with lysis buffer provided with the kit to make whole cell lysates, 20 μl of which was added to ELISA wells pre-coated with NFkB consensus DNA binding sequence. Bound NFkB was detected with antibody specific to the NFkB p50 subunit. Raji cell nuclear extract was used as a positive control. NFkB activation was expressed as absorbance at 450nm.

### Intracellular cytokine staining (ICS) assay

Frozen PBMC were thawed and seeded in 96 well plates at a density of 0.5–1 x 10^6^/well. Cells were activated with 10 μg/ml *Mtb* H37Rv lysate (BEI Resources, Manassas, VA, USA) and 5 μg/ml PHA (Remel, Thermo Fisher Scientific, Lenexa, KS, USA) for 16 hours. Along with stimulants, 1X monensin (BD Biosciences, USA) and 1X Brefeldin (BD Biosciences, USA) solution and 1 μg/ml BD Fast Immune CD28/49d (BD Biosciences, USA) were also added. Unstimulated cells were used as controls. At the end of 16 hrs, cells were first stained with an antibody cocktail comprising Avid, anti-CD45RA APC-H7, anti-CD127 PerCP Cy5.5, anti-HLA-DR FITC and anti-CD25 BV421 at 4°C for 10 minutes. Cells were then fixed with 1X FACS Lyse (BD Biosciences, USA), permeabilised with 1X FACS Perm (BD Biosciences, USA) and stained with an antibody cocktail of anti-CD3 BV570, anti-CD4 BUV395, anti-IFNγ Alexa Fluor 700 (clone B27, BD Pharmingen, BD Biosciences, USA), anti-IL-2 PE (clone MQ117H12, BD Biosciences, USA), anti-IL17A BV605 (clone BL168, Biolegend, San Diego, CA, USA), anti-IL-22 PECy7 (clone 22URT1, eBiosciences, San Diego, CA, USA) and anti-IL-10 BV786 (clone JES3-9D7, BD Biosciences, USA) at room temperature for 30 minutes. Cells were washed, fixed with 1% paraformaldehyde and acquired on BD FACS Aria Fusion using appropriate compensation controls. Data was analysed using FlowJo.

### Teff cell activation and RNA isolation for RNA sequencing

Sorted total and HLA-DR^-^ Teff cells were seeded at a density of 0.1 x 10^6^/well. To each well anti-CD3/anti-CD28 T cell activator beads were added at a bead:cell ratio of 1:1. Cells were cultured for 2, 24 and 96 hrs. At each time point cells were removed from wells, transferred to 1.5 ml tubes and pelleted at 3000 rpm for 5 min. The pellet was dissolved in 300 μl of TRIZOL Reagent (Ambion by Life Technologies, Carlsbad, CA, USA) except at the 0 hr time point where 50,000 cells were directly sorted into 1.2 ml of TRIZOL Reagent. RNA was isolated using the Direct-zol RNA Microprep Kit (ZYMO Research, Irvine, CA, USA) following the manufacturer’s instructions. Briefly, an equal of 100% ethanol (Merck, Darmstadt, Germany) was added to samples in TRIZOL and mixed. The samples were briefly spun to remove anti-CD3/anti-CD28 beads and the TRIZOL + ethanol mixture was loaded onto a Zymo-Spin IC column with collection tube and centrifuged at 10,000g for 30 seconds to allow the RNA to bind. An on-column DNA digestion was performed with DNase I (ZYMO Research, Irvine, CA, USA). Bound RNA was washed and eluted with 15 μl of RNase/DNase free water. Purified RNA was archived and used later either for sequencing or qRT-PCR.

### RNA Seq library construction and high-throughput sequencing

RNA sequencing library was prepared with Illumina-compatible SENSE mRNA-Seq Library Prep Kit V2 as per manufacturer’s recommendations for 10ng total RNA input (Lexogen, NH, USA) at Genotypic Technology Pvt. Ltd., Bangalore, India. Briefly, ~4-10ng of Qubit quantified RNA was taken for library prep. Poly (A) selection of the samples was performed using Oligo dT beads. The poly (A) RNA was subjected to reverse transcription and ligation followed by second strand synthesis. The double-stranded library was purified to remove magnetic beads and second strand synthesis reaction components. The library amplification was performed to add the complete adaptor sequences using 18 cycles of PCR for 10ng input and 22 cycles of PCR for <10ng input (initial denaturation at 98°C for 30s, cycling of 98°C for 10s, 65°C for 20s and 72°C for 30s, and final extension at 72°C for 1min). The final PCR product (sequencing library) was purified and subjected to library quality control check. The Illumina-compatible sequencing library was initially quantified by Qubit fluorometer (Thermo Fisher Scientific, MA, USA) and its fragment size distribution was analysed on Agilent TapeStation. Each index-coded library preparation was sequenced on an Illumina NextSeq 500 platform and 75 bp single-end (SE) reads were generated.

### Bioinformatic analysis of RNA-Seq data

The 75 bp single end reads generated by Illumina NextSeq 500 were quality checked and processed to remove the low-quality bases and the adapter contamination. The reads were aligned to the Homo sapiens GRCh38 reference taken from Ensemble (ftp://ftp.ensembl.org/pub/release77/fasta/homo_sapiens/dna/Homo_sapiens.GRCh38.dna.toplevel.fa.gz) using Tophat software (2.0.132). The input for the Tophat would be the processed data; parameters for directional libraries were included with other parameters in default. Transcript assembly was then done using Cufflinks-2.2.13 which assembles transcripts, estimates their abundances. Various assemblies generated from different samples are then merged together as a single assembly using cuffmerge script of cufflinks software. This software produces a single merged assembly in GTF format. Cuffdiff program of cufflinks software is used to find significant changes in transcript expression (DGEs). Merged GTF file produced by Cuffmerge was used as input for Cuffdiff. Differential gene expression studies were done between the combined control unstimulated and activated samples at all time points across all clinical categories. The genes which showed log_2_ fold change (FC) values less than -1 and p < 0.05 are represented as down-regulated, the genes which show an log_2_ FC greater than 1 and p < 0.05 were represented as up-regulated. Those with log_2_ fold change values between -1 and +1 were considered unregulated. For further analysis of the transcriptome data, a more stringent cut-off of log_2_ FC of > 2.5 for upregulated and < -2.5 for downregulated genes was applied for selection of biologically and functionally relevant genes. Differentially expressed genes (DEG) were enlisted for total Teff and HLA-DR^-^ Teff cells by comparing 0 hr and 2 hr, 24 hr and 96 hr gene expression. Next, genes differentially expressed with time were compared between the total Teff and HLA-DR^-^ Teff cells. DEGs were also subjected to pathway analysis using the DAVID Functional Annotation Bioinformatics Microarray Analysis tool.

### cDNA conversion and qRT-PCR

RNA was converted to cDNA using High capacity cDNA conversion kit (Applied Biosystems by Thermofisher Scientific, Vilnius, Lithuania). Selected genes were validated by qPCR using TaqMan Gene Expression Master Mix (Applied Biosystems, CA, USA) and TaqMan Gene Expression Assays for *GAPDH*, *CCL3L3*, *CCL4*, *PD-L1* (Thermo Fisher Scientific). GAPDH was used as the internal housekeeping control gene. All reactions were carried out in duplicate along with a no cDNA negative control using the Step One Plus (Applied Biosystems) instrument. Mean GAPDH C_T_ values were used for calculating relative copy number (RCN) of each gene.

### Statistical analysis

One-way ANOVA with Dunn’s multiple comparison test and Mann Whitney test were used for unpaired samples. Wilcoxon matched-pairs signed rank test was used for paired samples. Bonferroni correction was performed for multiple comparisons. All statistical analysis was carried out with the GraphPad Prism software.

## Supporting information

S1 Fig All clinical categories have comparable viability post activation. CFSE labeled sorted Teff cells were co-cultured with anti-CD3/anti-CD28 activator beads at a beads: cell ratio of 1:1. After 4 days, cells were stained with viability dye Avid. (A) A sequential gating strategy was used to obtain Avid^lo^CFSE^lo^ cells, which were considered as live proliferating cells. (B) Percentage of Avid^lo^CFSE^lo^ cells were plotted for N = 3 in each clinical category. Data shown is mean + SEM. P value was determined by non-parametric One-Way ANOVA Kruskal–Wallis test.
(PDF)

S2 Fig Treg mediated suppression of Teff cells varies across clinical categories. Memory Teff and Treg cells from different clinical categories were either sorted with magnetic beads (A) or flow cytometry (B). Teff cells were labeled with CFSE and co-cultured with different ratios of Treg cells in the presence of 2:1 (A) and 1:1 (B) anti-CD3/anti-CD28 activator beads. Proliferation was measured by CFSE dilution after 4 days of culture and percentage suppression was measured. Data shown is mean +/- SEM from multiple donors. Data was generated from N = 7 (A) and N = 4 (B) TB; N = 9 (A) and N = 6 (B) IGRA-ve; N = 4 (A) and N = 7 (B) IGRA+ve and N = 5 ATT (B) donors. P value was determined by non-parametric One-Way ANOVA Kruskal–Wallis test with Dunn’s multiple comparisons test. **p < 0.01, *p < 0.05.
(PDF)

S3 Fig Treg mediated suppression is lower in PTB CD4^+^ Teff cells activated with an antigen specific stimulus. Treg and PBMC minus Treg fractions were sorted with the help of flow cytometry. The PBMC minus Treg fraction was cultured alone (1:0) or along with autologous Treg at a 1:1 ratio. Cells were activated with 10 μg/ml *Mtb* lysate and IFNγ secretion was measured after 4 days by ELISA (A). Based upon levels of IFNγ in absence and presence of Treg cells, percent suppression was calculated (B). Data shown is median frequency/range from 10 PTB donors and 4 IGRA-ve donors. *P* value between paired samples was determined by Wilcoxon matched-pairs signed rank test and between unpaired by Mann Whitney test.
(PDF)

S4 Fig Expression of CD38 and PD-1 does not vary on Teff cells from different clinical categories. Thawed PBMC were stained with Avid, anti-CD3, anti-CD4, anti-CD45RA, anti-CD127, anti-CD25 anti-CD38 and anti-PD-1. Stained samples were acquired on a FACS Aria Fusion after using appropriate single color compensation controls. A sequential gating strategy was employed to arrive at live CD3^+^CD4^+^CD45RA^-^CD127^hi^CD25^lo^ Teff cells. Representative FACS plots of CD38^+^ (A) and PD-1^+^ (C) Teff cells from all clinical categories are shown. Teff frequencies of CD38^+^ (B) and PD-1^+^ (D) were calculated and plotted. Data shown is median frequency with range from multiple donors (IGRA-ve N = 9, IGRA+ve N = 11, PTB N = 27, ATT 6 months N = 8) in each clinical category. P value was determined by non-parametric One-Way ANOVA Kruskal–Wallis test.
(PDF)

S5 Fig Expression of HLA-DR, CD38 and PD-1 does not consistently vary on Treg cells from different clinical categories. Thawed PBMC were stained with Avid, anti-CD3, anti-CD4, anti-CD45RA, anti-CD127, anti-CD25, anti-HLA-DR, anti-CD38 and anti-PD-1. Stained samples were acquired on a FACS Aria Fusion after using appropriate single color compensation controls. A sequential gating strategy was employed to arrive at live CD3^+^CD4^+^CD45RA^-^CD127^lo^CD25^hi^ Treg cells. Frequencies of HLA-DR^+^ (A), CD38^+^ (B) and PD-1^+^(C) Treg cells were calculated and plotted. Data shown is median frequency with range from multiple donors (IGRA-ve N = 9, IGRA+ve N = 11, PTB N = 27, ATT 6 months N = 8) in each clinical category. P value was determined by non-parametric One-Way ANOVA Kruskal–Wallis test with Dunn’s multiple comparisons test. *p < 0.05.
(PDF)

S6 Fig HLA-DR^+^ Teff cells from PTB subjects are resistant to Treg mediated suppression. Sorted PTB total, HLA-DR^-^ and HLA-DR^+^ Teff cells were co-cultured with autologous Treg cells at a ratio of 1:1. Cells were activated with anti-CD3/anti-CD28 beads at beads: Teff cell ratio of 1:1. After 4 days, culture supernatants were collected and IFNγ was measured by ELISA. Percentage suppression was calculated based on IFNγ secretion in control cultures without Tregs and in cultures with Treg cells. Data shown is median frequency/range N = 4 for each cellular subset. P value was determined by non-parametric One-Way ANOVA Kruskal–Wallis test with Dunn’s multiple comparisons test. * p < 0.05.
(PDF)

S7 Fig Treg mediated suppression of *Mtb* specific responses is restored post depletion of HLA-DR^+^CD4^+^ T cells in PTB. Treg and PBMC minus Treg (denoted as total Teff) fractions were sorted with the help of flow cytometry from PTB donors. An additional subset of PBMCs depleted of Tregs and HLA-DR^+^CD4^+^ Teff (denoted as HLA-DR^-^ Teff) was also sorted from the same PTB donors. Total and HLA-DR^-^ Teff PBMC fractions were cultured alone (1:0) or along with autologous Treg at a 1:1 ratio. Cells were activated with 10 μg/ml *Mtb* lysate and IFNγ secretion was measured after 4 days by ELISA (A). Based upon levels of IFNγ in absence and presence of Treg cells, percent suppression was calculated (B). Data shown is median frequency/range from 6 donors each from PTB. *P* value between paired samples was determined by Wilcoxon matched-pairs signed rank test. *p < 0.05.
(PDF)

S8 Fig Gene expression across time. Total and HLA-DR^-^ Teff cells from PTB subjects were isolated by FACS and activated with anti-CD3/anti-CD28 activator beads (Teff:bead ratio of 1:1) for 2, 24 and 96 hrs. Unstimulated cells were used as 0 hr control. RNA was isolated at each time and subjected to RNA sequencing. Hierarchical clustering was done using FPKM values of genes (cut-off of log_2_-FC *≥ 2*.*5* for up and log_2_ FC ≤F-2.5 for downregulated genes, P < 0.05) using R software. Rows represent genes and columns represents samples. Distance matrix (dissimilarity between rows and columns) was calculated using euclidean distance method. Visualisations was done by calculating z score and adjusting colour. FPKM values from N = 5 total and N = 5 HLA-DR^-^ Teff samples were used at each time.
(PDF)

S9 Fig HLA-DR^-^ and HLA-DR^+^ Teff cells from PTB and IGRA-ve donors do not differ in their capacity to secrete IL-10. PBMC from PTB and IGRA-ve subjects were activated with either PHA or *Mtb* whole cell lysate. Brefeldin and monensin were added to cultures to prevent cytokine secretion. After 16 hrs of activation, cells were fixed, permeabilised and stained with an antibody cocktail comprising Avid, anti-CD3, anti-CD4, anti-CD45RA, anti-CD127, anti-CD25, anti-HLA-DR and anti-IL-10. Expression of IL-10 was measured in the Avid^-^CD3^+^CD4^+^CD45RA^-^CD127^hi^CD25^lo^HLA-DR^-^ and Avid^-^CD3^+^CD4^+^CD45RA^-^CD127^hi^CD25^lo^HLA-DR^+^ Teff compartments. A representative FACS plot of PHA activated PBMC and IL-10 secretion in HLA-DR^-^ and HLA-DR^+^ Teff fractions from PTB is shown (A). IL-10^+^ cells in response to stimulation were measured as a frequency of total and HLA-DR^-^ and HLA-DR^+^ Teff cells (B). A total of 9–11 subjects for PTB and 6 IGRA-ve were used.
(PDF)

S10 Fig Total and HLA-DR^-^ Teff cells from PTB donors differ in their capacity to secrete pro-inflammatory cytokines. PBMC from PTB subjects were activated with either PHA or *Mtb* whole cell lysate. Brefeldin and monensin were added to cultures to prevent cytokine secretion. After 16 hrs of activation, cells were fixed, permeabilised and stained with an antibody cocktail comprising Avid, anti-CD3, anti-CD4, anti-CD45RA, anti-CD127, anti-CD25, anti-HLA-DR, anti- IFNγ, anti-IL-2, anti-IL-17A and anti-IL-22. Expression of cytokines was measured in the Avid^-^CD3^+^CD4^+^CD45RA^-^CD127^hi^CD25^lo^ total and Avid^-^CD3^+^CD4^+^CD45RA^-^CD127^hi^CD25^lo^HLA-DR^-^ Teff compartments. A representative FACS plot of PHA activated PBMC and cytokine expression in total and HLA-DR^-^ Teff fractions is shown (A). IFNγ^+^, IL-2^+^, IL-17A^+^ and IL-22^+^ cells in response to stimulation were measured as a frequency of total and HLA-DR^-^ Teff cells (B). A total of 9 subjects for PHA and 11 each for *Mtb* lysate were used. Paired Wilcoxon matched-pairs signed rank test with Bonferroni correction for multiple comparison was used to determine *P* value. *p ≤ 0.013.
(PDF)

S11 Fig HLA-DR^+^CD4^+^ memory T cells from PTB subjects produce more pro-inflammatory cytokines compared to HLA-DR^+^CD4^+^ memory T cells from IGRA-ve individuals. PBMC from IGRA-ve and PTB subjects were activated with either PHA or *Mtb* whole cell lysate. Brefeldin and monensin were added to cultures to prevent cytokine secretion. After 16 hrs of activation, cells were fixed, permeabilised and stained with an antibody cocktail comprising Avid, anti-CD3, anti-CD4, anti-CD45RA, anti-CD127, anti-CD25, anti-HLA-DR, anti-IFNγ, anti-IL-2, anti-IL-17A and anti-IL-22. Expression of cytokines (IFNγ, IL-2, IL-17A and IL-22) was measured in the Avid^-^CD3^+^CD4^+^CD45RA^-^CD127^hi^CD25^lo^HLA-DR^+^ Teff compartments. Frequencies of cytokine producing HLA-DR^+^ Teff cells from IGRA-ve and PTB subjects were compared. Upper panel shows data pertaining to PHA stimulation and lower panel shows data pertaining to *Mtb* lysate stimulation. Data shown is median frequency/range from 6 IGRA-ve and 9–11 PTB individuals. P value was determined by Mann-Whitney test. **p < 0.01, *p < 0.05.
(PDF)

S12 Fig Signaling through CCR5 and PD-L1 receptors is intact in total and HLA-DR^-^ Teff subsets from IGRA-ve subjects. Sorted IGRA-ve total and HLA-DR^-^ Teff cells were treated with 50 ng/ml each of CCL3 and CCL4 (A), 5 μg/ml anti-CCR5 (A), 5 μg/ml anti-PD-L1 (B) or 10 μg/ml recombinant PD-L1 (B). Cells were activated with anti-CD3/anti-CD28 beads (1:1 ratio). Anti-CCR5, anti-PD-L1 and recombinant PD-L1 were added 20 minutes prior to addition of mitogenic anti-CD3/anti-CD28. Unstimulated cells were used as control. NFκB activation was measured by ELISA after 180 minutes of stimulation and was expressed as absorbance at 450 nm. Cells from a total of N = 3 IGRA-ve donors were used for the assay. (A) shows data pertaining to CCR5 mediated signaling and (B) shows data pertaining to PD-L1 mediated signaling. Data shown is mean + SEM.
(PDF)

S1 Table Detailed clinical information of enrolled subjects. (XLS)

S2 Table Comprehensive list of DEGs in activated PTB total and HLA-DR^-^ Teff cells over time. Total and HLA-DR^-^ Teff cells from PTB subjects were isolated by FACS and activated with anti-CD3/anti-CD28 activator beads (Teff: bead ratio of 1:1) for 2, 24 and 96 hrs. A comprehensive list of up and down-regulated DEGs including isoforms (cut-off of log_2_ FC ≥ 2.5/log_2_ FC ≤ -2.5, P < 0.05) was obtained by comparing gene expression post activation to that at 0 hr in both total and HLA-DR^-^ Teff cells.
(XLSX)
