# Supplementary figures and images for: Circulating HLA-DR+CD4+ effector memory T cells resistant to CCR5 and PD-L1 mediated suppression compromise regulatory T cell function in tuberculosis
Source: PLoS Pathog. 2018 Sep 19;14(9):e1007289. doi: 10.1371/journal.ppat.1007289 (PMC6166982; doi:10.1371/journal.ppat.1007289)

(A)

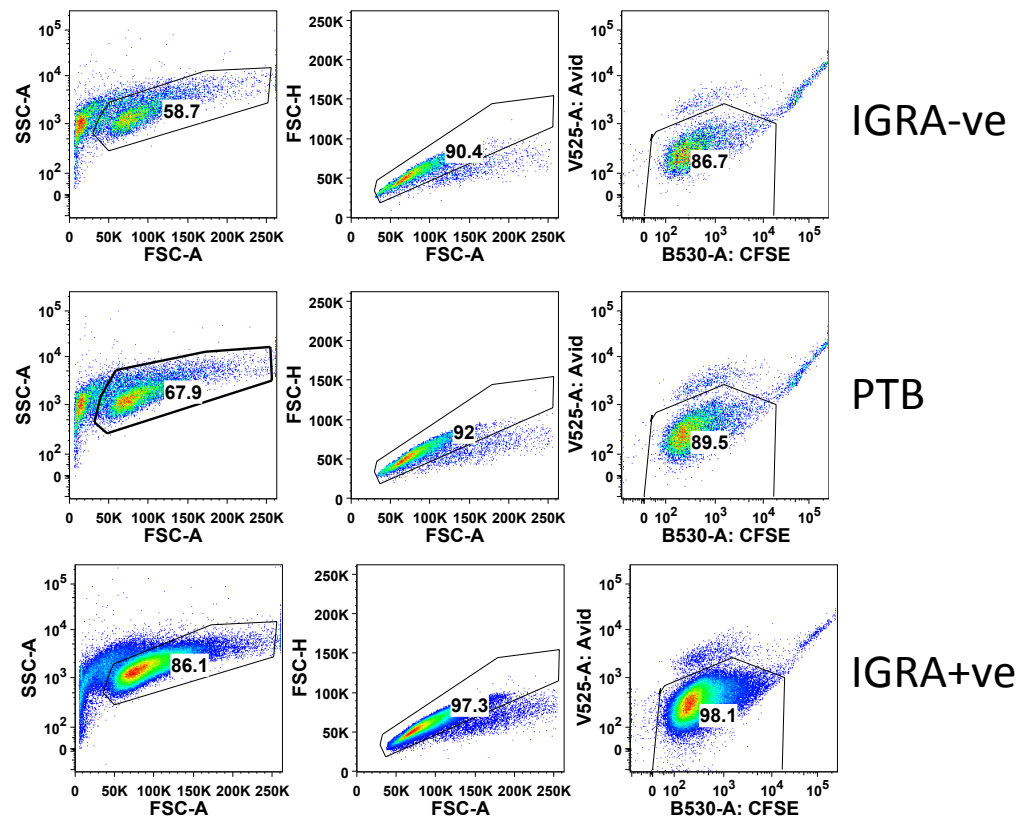

(B)

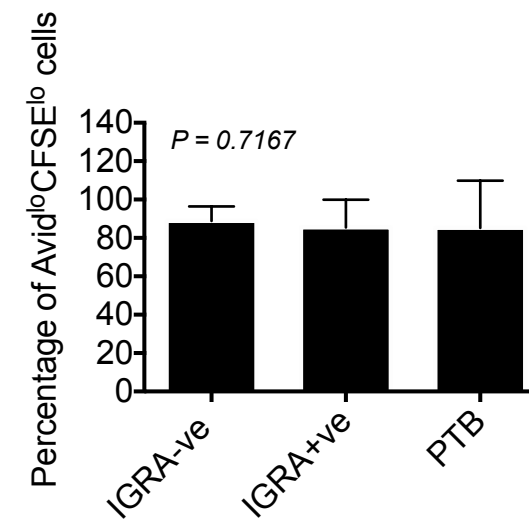

Supplement: S1 Fig — CFSE labeled sorted Teff cells were co-cultured with anti-CD3/anti-CD28 activator beads at a beads: cell ratio of 1:1. After 4 days, cells were stained with viability dye Avid. (A) A sequential gating strategy was used to obtain AvidloCFSElo cells, which were considered as live proliferating cells. (B) Percentage of AvidloCFSElo cells were plotted for N = 3 in each clinical category. Data shown is mean + SEM. P value was determined by non-parametric One-Way ANOVA Kruskal–Wallis test. (PDF) [file ppat.1007289.s001.pdf]

(A)

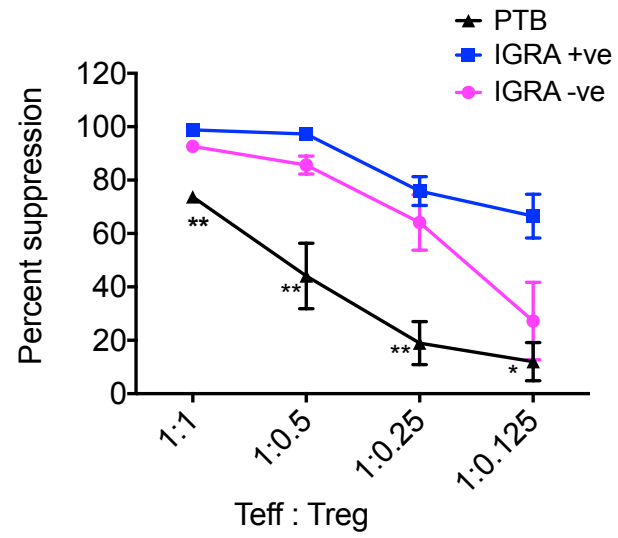

(B)

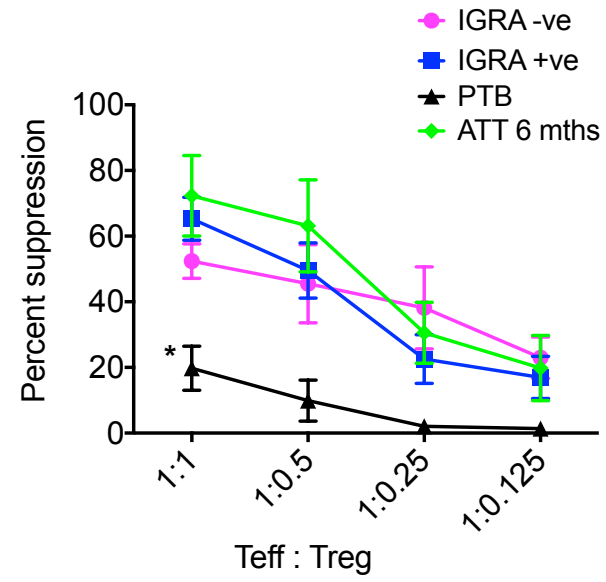

Supplement: S2 Fig — Memory Teff and Treg cells from different clinical categories were either sorted with magnetic beads (A) or flow cytometry (B). Teff cells were labeled with CFSE and co-cultured with different ratios of Treg cells in the presence of 2:1 (A) and 1:1 (B) anti-CD3/anti-CD28 activator beads. Proliferation was measured by CFSE dilution after 4 days of culture and percentage suppression was measured. Data shown is mean +/- SEM from multiple donors. Data was generated from N = 7 (A) and N = 4 (B) TB; N = 9 (A) and N = 6 (B) IGRA-ve; N = 4 (A) and N = 7 (B) IGRA+ve and N = 5 ATT (B) donors. P value was determined by non-parametric One-Way ANOVA Kruskal–Wallis test with Dunn’s multiple comparisons test. **p < 0.01, *p < 0.05. (PDF) [file ppat.1007289.s002.pdf]

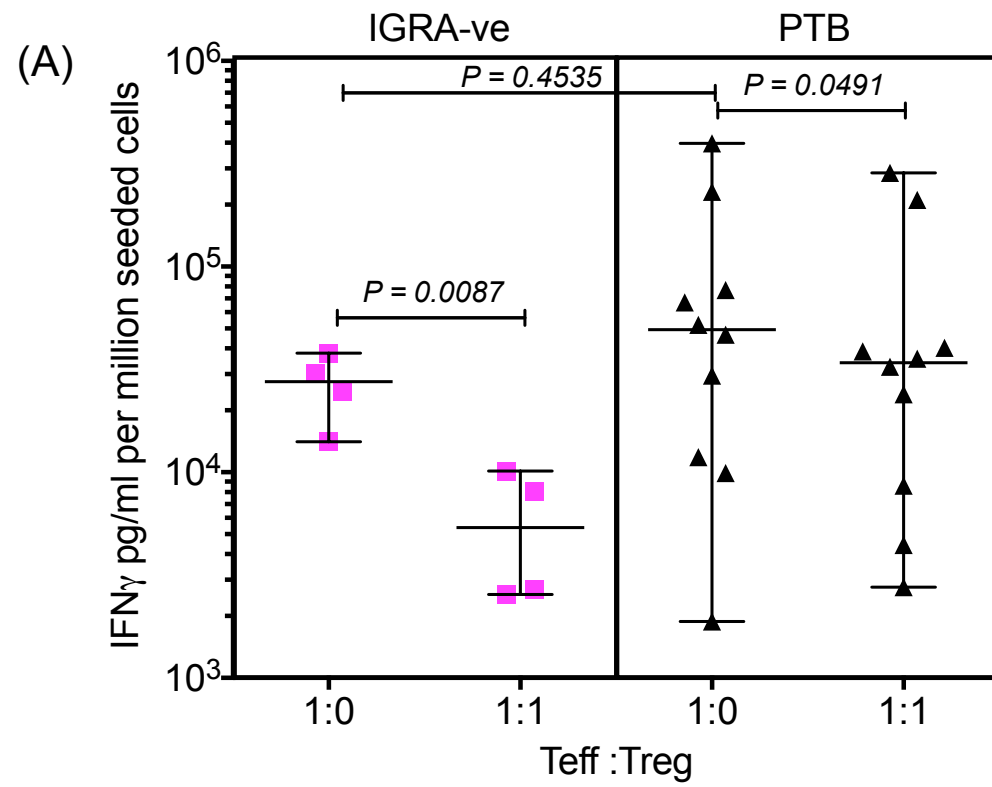

(B)

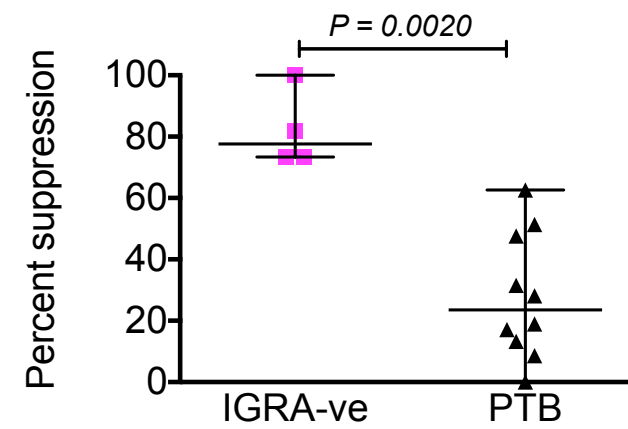

Supplement: S3 Fig — Treg and PBMC minus Treg fractions were sorted with the help of flow cytometry. The PBMC minus Treg fraction was cultured alone (1:0) or along with autologous Treg at a 1:1 ratio. Cells were activated with 10 μg/ml Mtb lysate and IFNγ secretion was measured after 4 days by ELISA (A). Based upon levels of IFNγ in absence and presence of Treg cells, percent suppression was calculated (B). Data shown is median frequency/range from 10 PTB donors and 4 IGRA-ve donors. P value between paired samples was determined by Wilcoxon matched-pairs signed rank test and between unpaired by Mann Whitney test. (PDF) [file ppat.1007289.s003.pdf]

(A) IGRA-ve IGRA+ve PTB ATT (B)

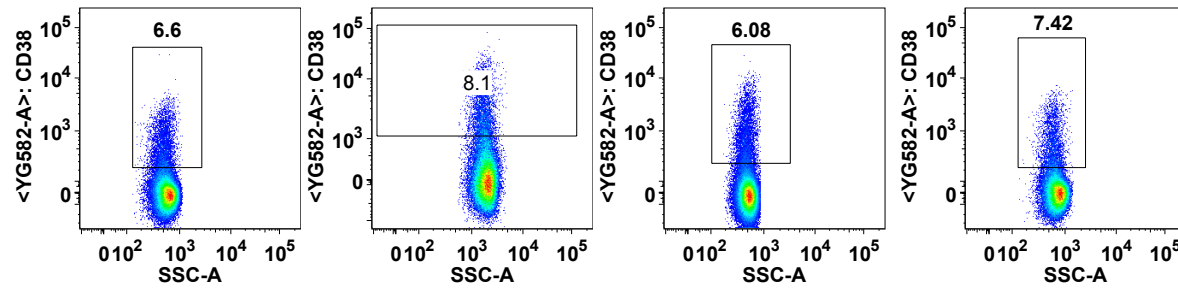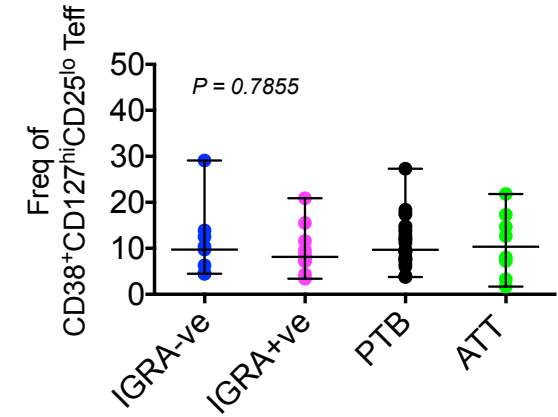

(C) IGRA-ve IGRA+ve PTB ATT (D)

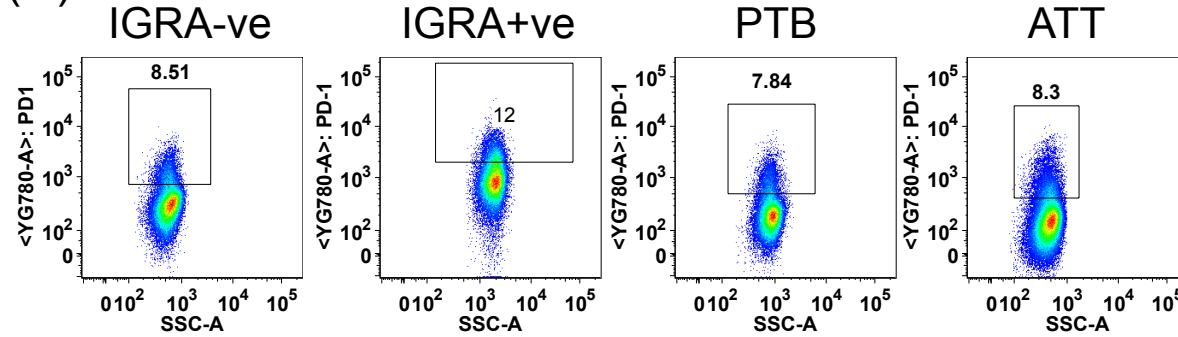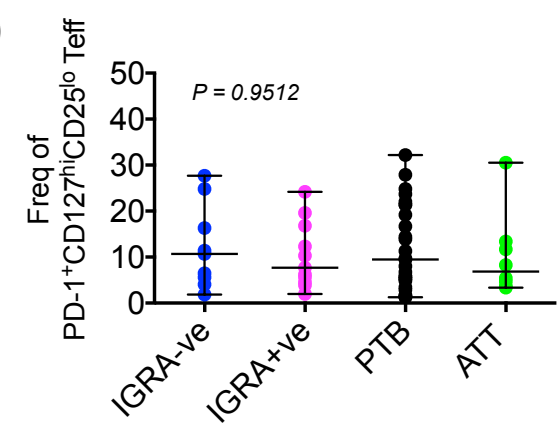

Supplement: S4 Fig — Thawed PBMC were stained with Avid, anti-CD3, anti-CD4, anti-CD45RA, anti-CD127, anti-CD25 anti-CD38 and anti-PD-1. Stained samples were acquired on a FACS Aria Fusion after using appropriate single color compensation controls. A sequential gating strategy was employed to arrive at live CD3+CD4+CD45RA-CD127hiCD25lo Teff cells. Representative FACS plots of CD38+ (A) and PD-1+ (C) Teff cells from all clinical categories are shown. Teff frequencies of CD38+ (B) and PD-1+ (D) were calculated and plotted. Data shown is median frequency with range from multiple donors (IGRA-ve N = 9, IGRA+ve N = 11, PTB N = 27, ATT 6 months N = 8) in each clinical category. P value was determined by non-parametric One-Way ANOVA Kruskal–Wallis test. (PDF) [file ppat.1007289.s004.pdf]

(A)

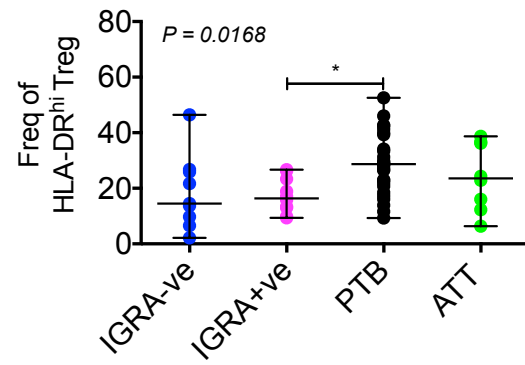

(B)

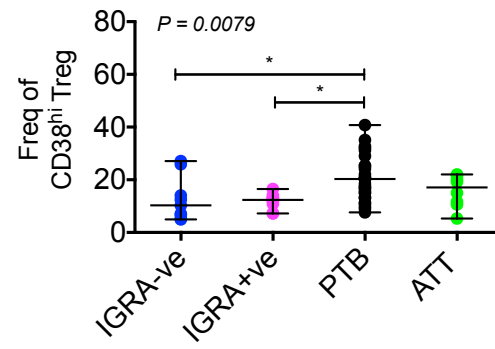

(C)

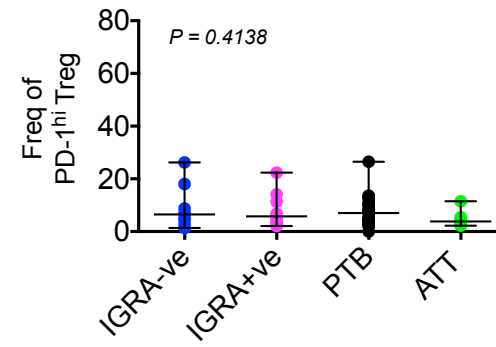

Supplement: S5 Fig — Thawed PBMC were stained with Avid, anti-CD3, anti-CD4, anti-CD45RA, anti-CD127, anti-CD25, anti-HLA-DR, anti-CD38 and anti-PD-1. Stained samples were acquired on a FACS Aria Fusion after using appropriate single color compensation controls. A sequential gating strategy was employed to arrive at live CD3+CD4+CD45RA-CD127loCD25hi Treg cells. Frequencies of HLA-DR+ (A), CD38+ (B) and PD-1+(C) Treg cells were calculated and plotted. Data shown is median frequency with range from multiple donors (IGRA-ve N = 9, IGRA+ve N = 11, PTB N = 27, ATT 6 months N = 8) in each clinical category. P value was determined by non-parametric One-Way ANOVA Kruskal–Wallis test with Dunn’s multiple comparisons test. *p < 0.05. (PDF) [file ppat.1007289.s005.pdf]

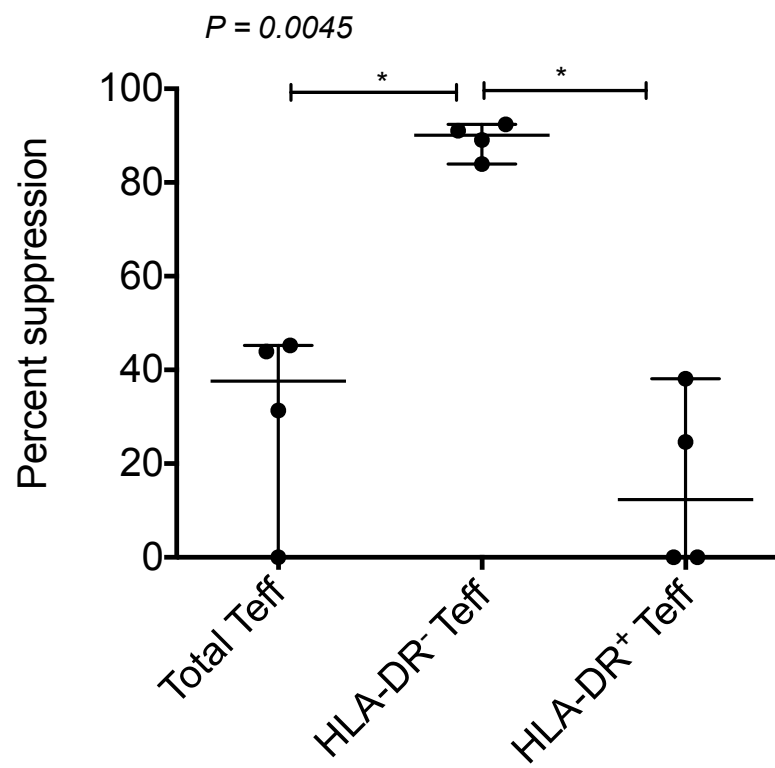

Supplement: S6 Fig — Sorted PTB total, HLA-DR- and HLA-DR+ Teff cells were co-cultured with autologous Treg cells at a ratio of 1:1. Cells were activated with anti-CD3/anti-CD28 beads at beads: Teff cell ratio of 1:1. After 4 days, culture supernatants were collected and IFNγ was measured by ELISA. Percentage suppression was calculated based on IFNγ secretion in control cultures without Tregs and in cultures with Treg cells. Data shown is median frequency/range N = 4 for each cellular subset. P value was determined by non-parametric One-Way ANOVA Kruskal–Wallis test with Dunn’s multiple comparisons test. * p < 0.05. (PDF) [file ppat.1007289.s006.pdf]

(A)

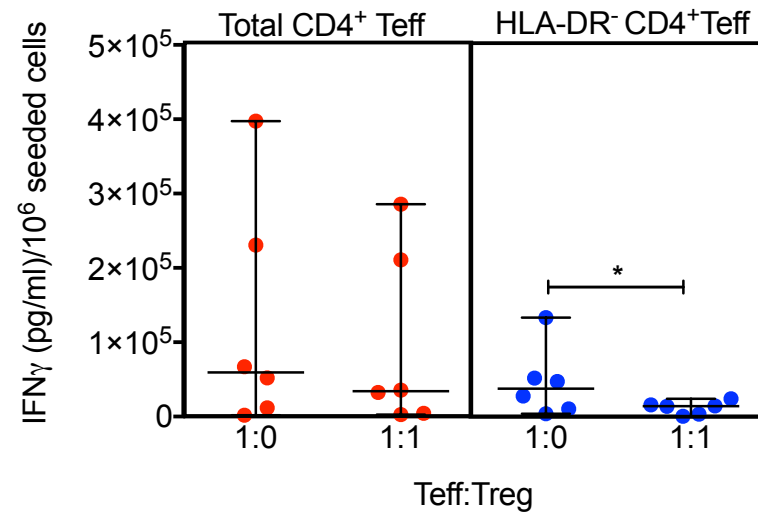

(B)

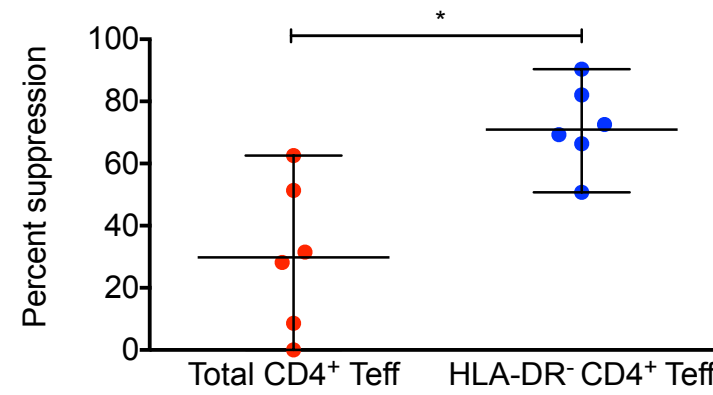

Supplement: S7 Fig — Treg and PBMC minus Treg (denoted as total Teff) fractions were sorted with the help of flow cytometry from PTB donors. An additional subset of PBMCs depleted of Tregs and HLA-DR+CD4+ Teff (denoted as HLA-DR- Teff) was also sorted from the same PTB donors. Total and HLA-DR- Teff PBMC fractions were cultured alone (1:0) or along with autologous Treg at a 1:1 ratio. Cells were activated with 10 μg/ml Mtb lysate and IFNγ secretion was measured after 4 days by ELISA (A). Based upon levels of IFNγ in absence and presence of Treg cells, percent suppression was calculated (B). Data shown is median frequency/range from 6 donors each from PTB. P value between paired samples was determined by Wilcoxon matched-pairs signed rank test. *p < 0.05. (PDF) [file ppat.1007289.s007.pdf]

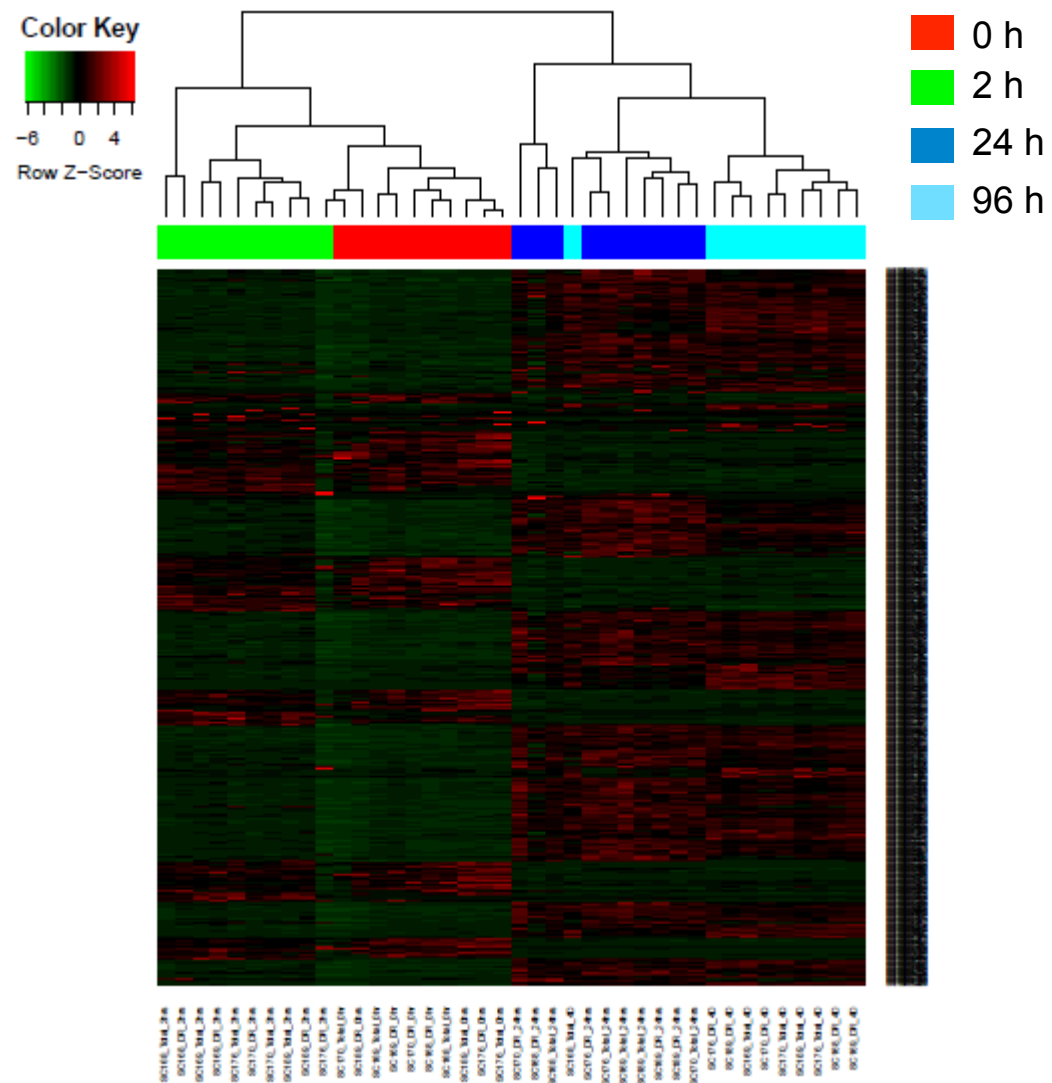

Supplement: S8 Fig — Total and HLA-DR- Teff cells from PTB subjects were isolated by FACS and activated with anti-CD3/anti-CD28 activator beads (Teff:bead ratio of 1:1) for 2, 24 and 96 hrs. Unstimulated cells were used as 0 hr control. RNA was isolated at each time and subjected to RNA sequencing. Hierarchical clustering was done using FPKM values of genes (cut-off of log2-FC ≥ 2.5 for up and log2 FC ≤F-2.5 for downregulated genes, P < 0.05) using R software. Rows represent genes and columns represents samples. Distance matrix (dissimilarity between rows and columns) was calculated using euclidean distance method. Visualisations was done by calculating z score and adjusting colour. FPKM values from N = 5 total and N = 5 HLA-DR- Teff samples were used at each time. (PDF) [file ppat.1007289.s008.pdf]

(A)

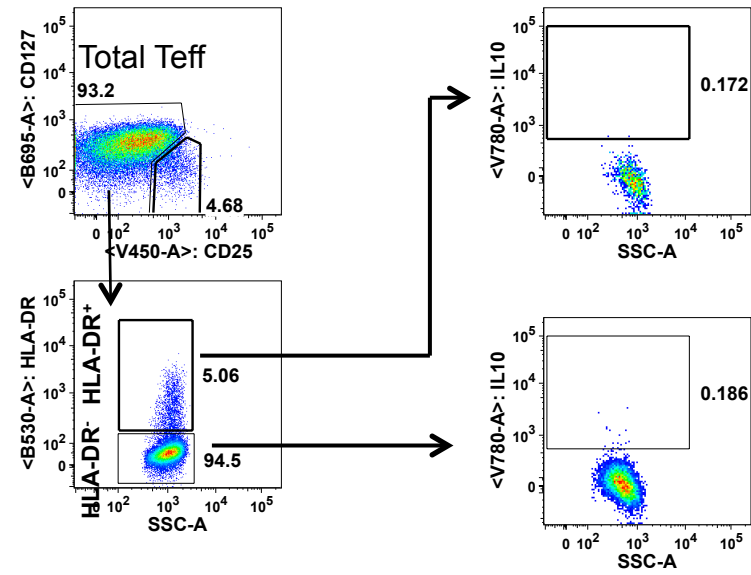

PHA

*Mtb* lysate

(B)

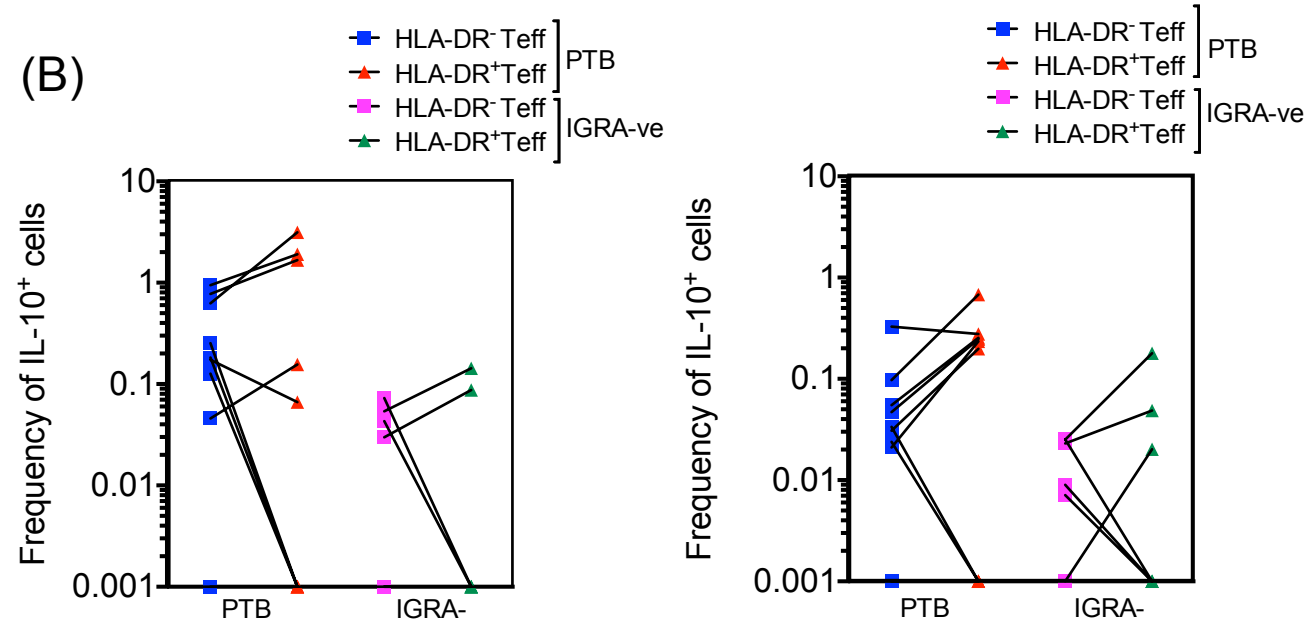

Supplement: S9 Fig — PBMC from PTB and IGRA-ve subjects were activated with either PHA or Mtb whole cell lysate. Brefeldin and monensin were added to cultures to prevent cytokine secretion. After 16 hrs of activation, cells were fixed, permeabilised and stained with an antibody cocktail comprising Avid, anti-CD3, anti-CD4, anti-CD45RA, anti-CD127, anti-CD25, anti-HLA-DR and anti-IL-10. Expression of IL-10 was measured in the Avid-CD3+CD4+CD45RA-CD127hiCD25loHLA-DR- and Avid-CD3+CD4+CD45RA-CD127hiCD25loHLA-DR+ Teff compartments. A representative FACS plot of PHA activated PBMC and IL-10 secretion in HLA-DR- and HLA-DR+ Teff fractions from PTB is shown (A). IL-10+ cells in response to stimulation were measured as a frequency of total and HLA-DR- and HLA-DR+ Teff cells (B). A total of 9–11 subjects for PTB and 6 IGRA-ve were used. (PDF) [file ppat.1007289.s009.pdf]

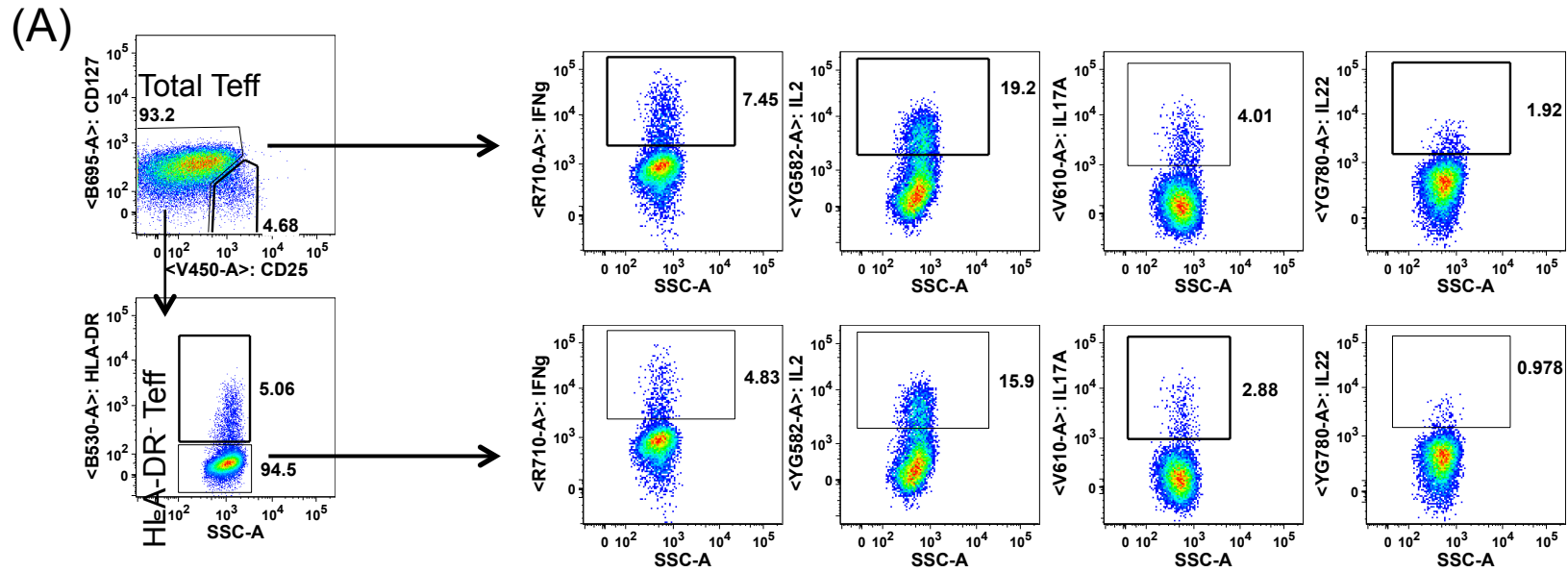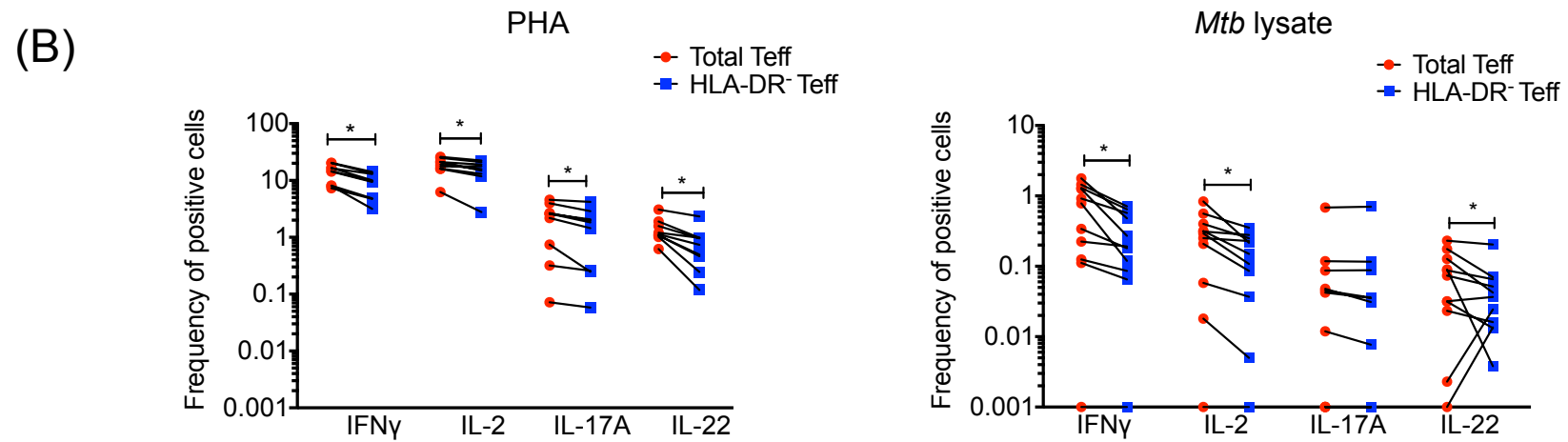

Supplement: S10 Fig — PBMC from PTB subjects were activated with either PHA or Mtb whole cell lysate. Brefeldin and monensin were added to cultures to prevent cytokine secretion. After 16 hrs of activation, cells were fixed, permeabilised and stained with an antibody cocktail comprising Avid, anti-CD3, anti-CD4, anti-CD45RA, anti-CD127, anti-CD25, anti-HLA-DR, anti- IFNγ, anti-IL-2, anti-IL-17A and anti-IL-22. Expression of cytokines was measured in the Avid-CD3+CD4+CD45RA-CD127hiCD25lo total and Avid-CD3+CD4+CD45RA-CD127hiCD25loHLA-DR- Teff compartments. A representative FACS plot of PHA activated PBMC and cytokine expression in total and HLA-DR- Teff fractions is shown (A). IFNγ+, IL-2+, IL-17A+ and IL-22+ cells in response to stimulation were measured as a frequency of total and HLA-DR- Teff cells (B). A total of 9 subjects for PHA and 11 each for Mtb lysate were used. Paired Wilcoxon matched-pairs signed rank test with Bonferroni correction for multiple comparison was used to determine P value. *p ≤ 0.013. (PDF) [file ppat.1007289.s010.pdf]

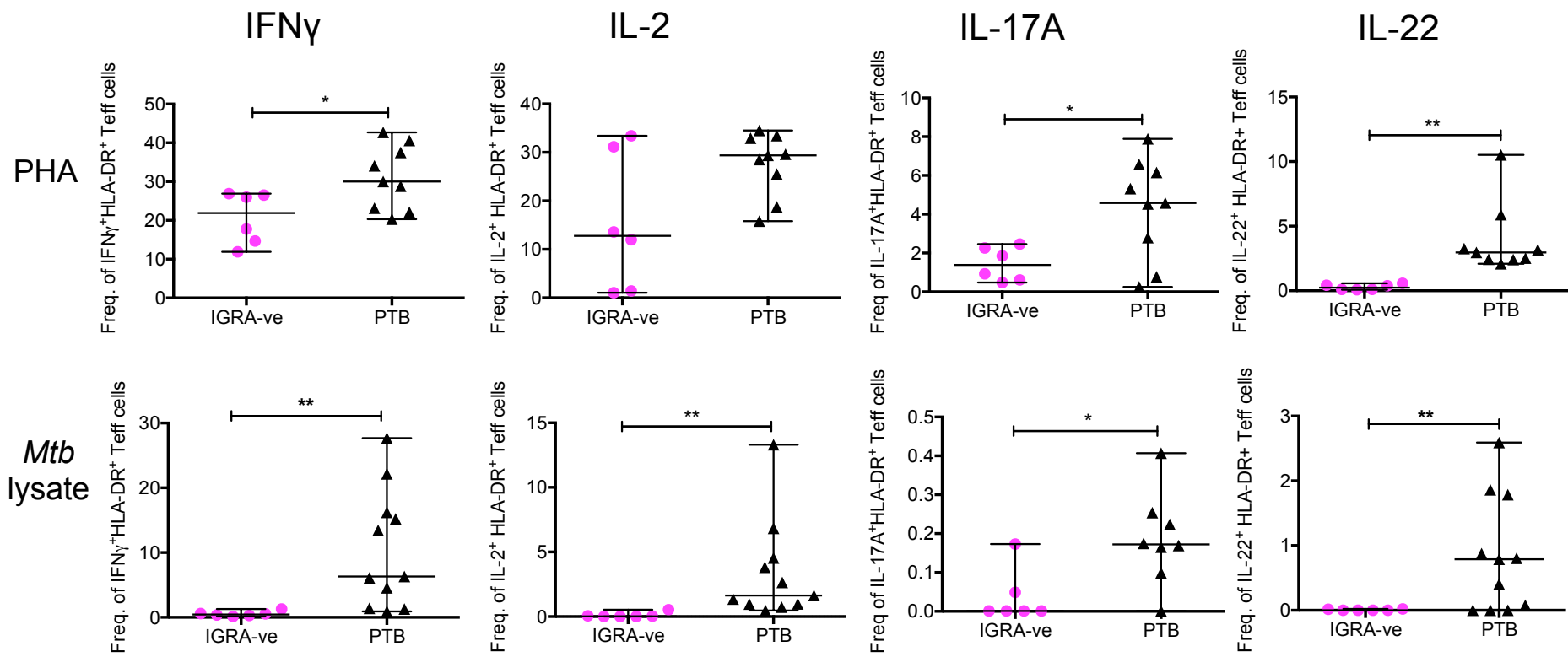

Supplement: S11 Fig — PBMC from IGRA-ve and PTB subjects were activated with either PHA or Mtb whole cell lysate. Brefeldin and monensin were added to cultures to prevent cytokine secretion. After 16 hrs of activation, cells were fixed, permeabilised and stained with an antibody cocktail comprising Avid, anti-CD3, anti-CD4, anti-CD45RA, anti-CD127, anti-CD25, anti-HLA-DR, anti-IFNγ, anti-IL-2, anti-IL-17A and anti-IL-22. Expression of cytokines (IFNγ, IL-2, IL-17A and IL-22) was measured in the Avid-CD3+CD4+CD45RA-CD127hiCD25loHLA-DR+ Teff compartments. Frequencies of cytokine producing HLA-DR+ Teff cells from IGRA-ve and PTB subjects were compared. Upper panel shows data pertaining to PHA stimulation and lower panel shows data pertaining to Mtb lysate stimulation. Data shown is median frequency/range from 6 IGRA-ve and 9–11 PTB individuals. P value was determined by Mann-Whitney test. **p < 0.01, *p < 0.05. (PDF) [file ppat.1007289.s011.pdf]

(A)

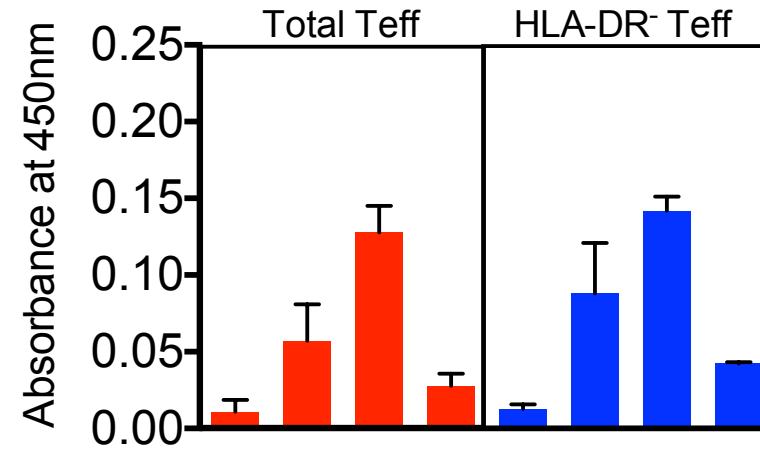

|                    |   |   |   |   |   |   |   |   |
|--------------------|---|---|---|---|---|---|---|---|
| CCL3 + CCL4        | - | + | - | - | - | + | - | - |
| anti-CCR5          | - | - | - | + | - | - | - | + |
| anti-CD3/anti-CD28 | - | - | + | + | - | - | + | + |

(B)

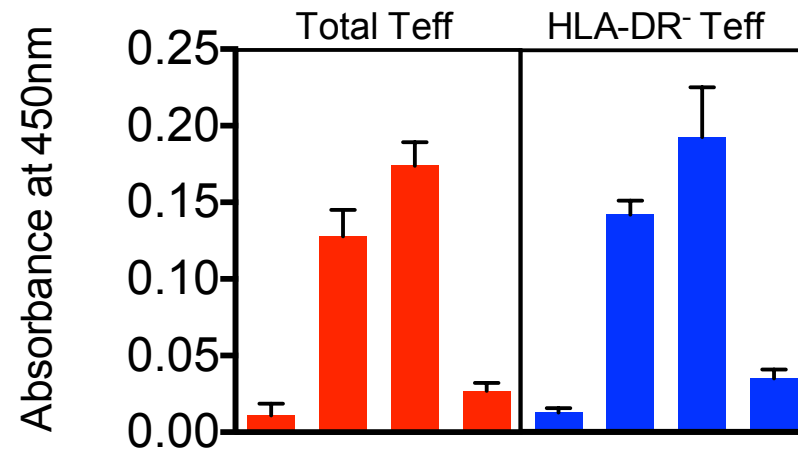

|                    |   |   |   |   |   |   |   |   |
|--------------------|---|---|---|---|---|---|---|---|
| anti-CD3/anti-CD28 | - | + | + | + | - | + | + | + |
| anti-PD-L1         | - | - | + | - | - | - | + | - |
| recombinant PD-L1  | - | - | - | + | - | - | - | + |

Supplement: S12 Fig — Sorted IGRA-ve total and HLA-DR- Teff cells were treated with 50 ng/ml each of CCL3 and CCL4 (A), 5 μg/ml anti-CCR5 (A), 5 μg/ml anti-PD-L1 (B) or 10 μg/ml recombinant PD-L1 (B). Cells were activated with anti-CD3/anti-CD28 beads (1:1 ratio). Anti-CCR5, anti-PD-L1 and recombinant PD-L1 were added 20 minutes prior to addition of mitogenic anti-CD3/anti-CD28. Unstimulated cells were used as control. NFκB activation was measured by ELISA after 180 minutes of stimulation and was expressed as absorbance at 450 nm. Cells from a total of N = 3 IGRA-ve donors were used for the assay. (A) shows data pertaining to CCR5 mediated signaling and (B) shows data pertaining to PD-L1 mediated signaling. Data shown is mean + SEM. (PDF) [file ppat.1007289.s012.pdf]
